# Synthetic growth by self-lubricated photopolymerization and extrusion inspired by plants and fungi

**DOI:** 10.1073/pnas.2201776119

**Published:** 2022-08-09

**Authors:** Matthew M. Hausladen, Boran Zhao, Matthew S. Kubala, Lorraine F. Francis, Timothy M. Kowalewski, Christopher J. Ellison

**Affiliations:** ^a^Department of Chemical Engineering and Materials Science, University of Minnesota, Minneapolis, MN 55455;; ^b^Department of Mechanical Engineering, University of Minnesota, Minneapolis, MN 55455

**Keywords:** synthetic growth, photopolymerization, soft robotics, extrusion, biomimetics

## Abstract

Many natural organisms, such as fungal hyphae and plant roots, grow at their tips, enabling the generation of complex bodies composed of natural materials as well as dexterous movement and exploration. Tip growth presents an exemplary process by which materials synthesis and actuation are coupled, providing a blueprint for how growth could be realized in a synthetic system. Herein, we identify three underlying principles essential to tip-based growth of biological organisms: a fluid pressure driving force, localized polymerization for generating structure, and fluid-mediated transport of constituent materials. In this work, these evolved features inspire a synthetic materials growth process called extrusion by self-lubricated interface photopolymerization (E-SLIP), which can continuously fabricate solid profiled polymer parts with tunable mechanical properties from liquid precursors. To demonstrate the utility of E-SLIP, we create a tip-growing soft robot, outline its fundamental governing principles, and highlight its capabilities for growth at speeds up to 12 cm/min and lengths up to 1.5 m. This growing soft robot is capable of executing a range of tasks, including exploration, burrowing, and traversing tortuous paths, which highlight the potential for synthetic growth as a platform for on-demand manufacturing of infrastructure, exploration, and sensing in a variety of environments.

## Significance

Growth in nature often couples material generation and actuation, offering an intriguing paradigm for the marriage of materials science and robotics. Inspired by the growth of plants and fungi, a new approach for synthetic materials growth was developed based on simultaneous self-lubricated photopolymerization and extrusion. This strategy enables a new continuous method for light-based fabrication of profiled parts not possible with state-of-the-art three-dimensional (3D) printing or other methods. We exploit this materials growth paradigm to produce a soft robot capable of rapid continuous growth, thereby addressing major limitations of growing soft robots that stem from limited extensibility, lack of permanent structure, and inability to negotiate torturous paths, demonstrating the potential of growth to provide new capabilities in manufacturing and soft robotics.

**Fig. 1.**
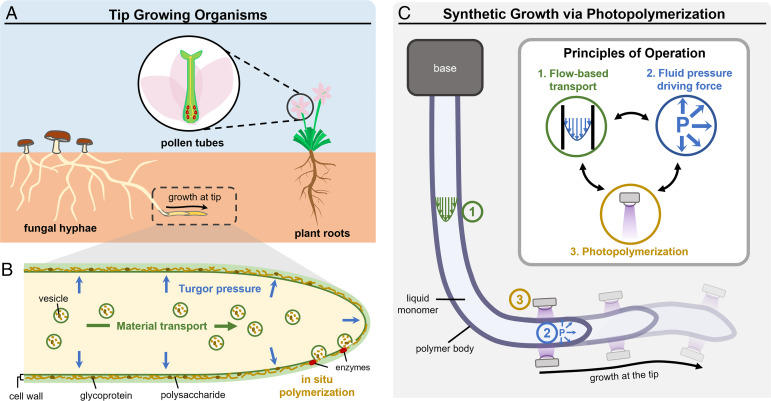
Synthetic growth method inspired by principles of biological tip growth. (*A*) Plant roots, fungal hyphae, and pollen tubes exemplify tip-growing organisms, which lengthen their bodies over time, allowing them to avoid obstacles, and have reduced friction with the environment since only the tip is in relative motion with the environment. (*B*) Three key principles of tip growth appear across biological kingdoms: a turgor pressure driving force, local solid structure generation at the tip through in situ polymerization, and fluid-mediated material transport. (*C*) Synthetic growth concept inspired by the key principles of tip growth, with photopolymerization building solid structure locally and monomer solution used to generate pressure (P) for growth and transport material.

Diverse creatures and cells across biological kingdoms, such as plant roots, fungal hyphae, and pollen tubes, leverage a particular method of growth, known as tip growth, as a strategy to navigate and interact with their environment (1). Tip growth is characterized by anisotropic addition of new material at the growing end of a body, with only the tip in motion relative to the environment (Fig. 1*A*). This localized growth greatly reduces the resistance imposed by the surroundings and permits an agile response to environmental conditions. Therefore, tip-growing organisms are able to generate large complex structures over time, traverse constrained environments, such as soil or biological tissue (2, 3), and navigate according to environmental stimuli, such as light (4), chemical gradients (5), or mechanical impedance (6).

Scientists and engineers have often looked to nature for inspiration for the next generation of materials (7–9) and robots (10–14), with particular interest toward soft robots composed of compliant materials that mimic living tissues and organism motions. In this vein, pioneering work has sought to translate the benefits of tip growth into engineered systems. These works utilized the pressurized eversion of a thin polymer film (15) or a filament-based additive manufacturing process (16) to build or extend structure in a manner akin to tip growth. However, both these designs rely on a continuous, solid-state supply of building material, which leads to a rapid rise in internal friction during growth on tortuous paths, limiting ultimate extension. Therefore, we sought to highlight and leverage known principles of tip growth found in natural systems, which produce structures with substantial tortuosity (17, 18), to overcome these limitations in a synthetic analog.

The mechanisms of tip growth in the well-studied cases of fungal hyphae (19), root tips (5), and pollen tubes (20) share several underlying principles (Fig. 1 *A* and *B*). The first principle is that a major driver of growth is fluid pressure. This pressure is thought to arise from the internal turgor pressure within cells, which is generated by an osmotic potential between the fluid-filled cell and its environment. As internal pressure deforms extensible cell walls, their selective yielding at the tip accommodates growth. The second principle is that growth occurs through localized cell wall synthesis. Cell wall components, namely polysaccharides, such as chitin in fungi, cellulose in algae and plants, and glycoproteins, are polymerized at the tip to locally build solid structure (21, 22). Lastly, the third principle is fluid-mediated material transport, in which cell wall components are transported to the tip by both flow-based and active means (such as vesicular transport via the cytoskeleton) (23). By combining these three principles, organisms are able to generate large forces and lengthen at the tip with minimal friction with their surroundings (24).

We sought to emulate these biological principles in a synthetic system of tip growth in analogy to the capabilities evolved by nature. In particular, photopolymerization offers a unique approach for the localized buildup of solid structure that is central to tip growth. Photopolymerization permits the generation of three-dimensional (3D) polymer objects from a liquid resin, with spatial and temporal control over the mechanical and chemical properties of produced objects (25–27). Due to these advantages, it has provided new capabilities in manufacturing technologies, such as light-based additive manufacturing (28, 29), soft lithography (30), fiber processing (31–33), and flow lithography (34). In our synthetic analog of tip growth, photopolymerization enables the local and rapid polymerization of the structure, while pressure-driven flow is utilized to supply liquid monomer solution to the site of photopolymerization at the tip, thus driving growth (Fig. 1*C*). As an example of the usefulness of synthetic growth, we implement this strategy in a soft robot capable of replicating the biological functionalities of tip-growing organisms.

## Results and Discussion

**Fig. 2.**
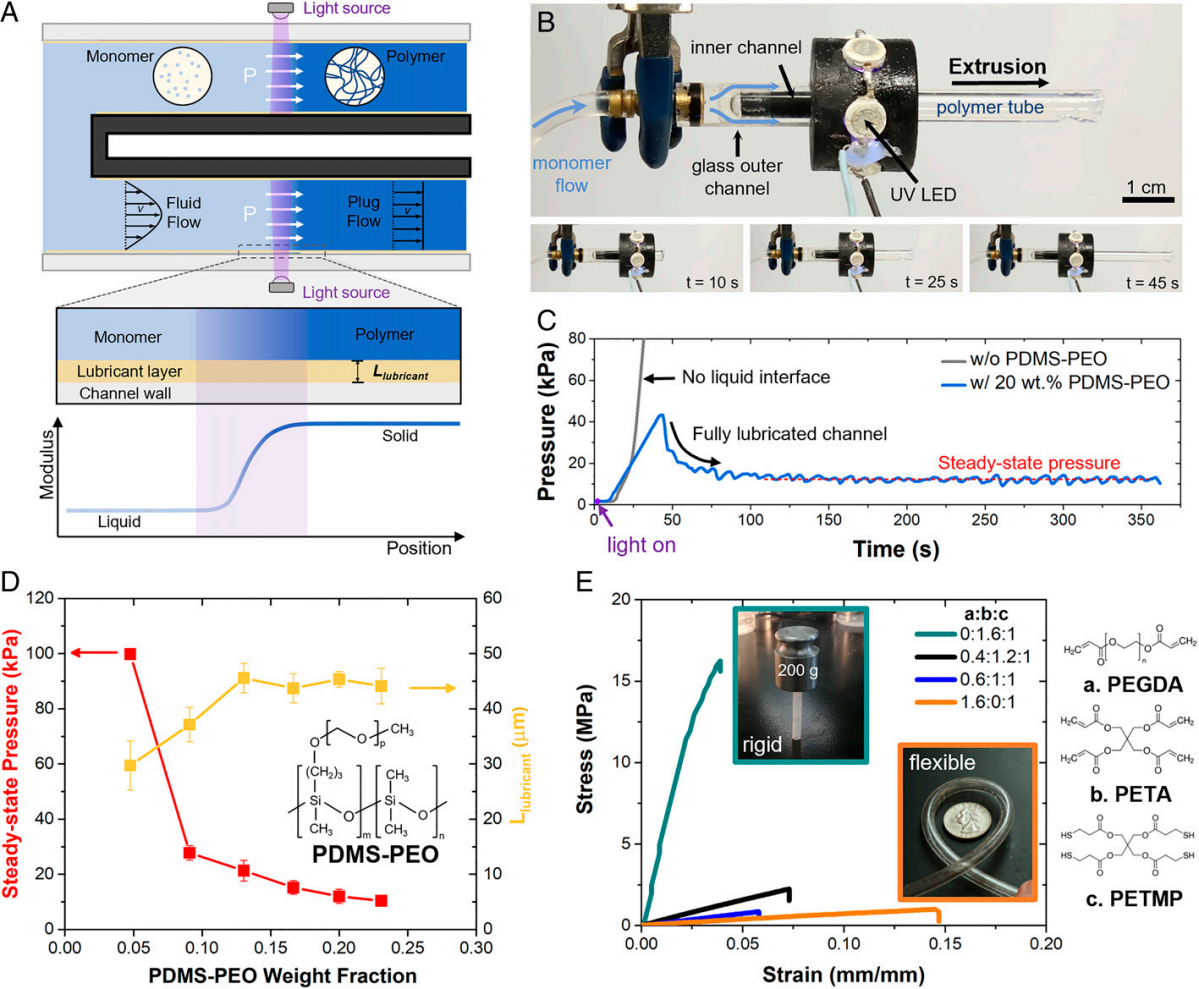
Extrusion by self-lubricated interface photopolymerization (E-SLIP). (*A*) Schematic demonstrating the E-SLIP process: continuous photopolymerization of a pressure-driven flow of liquid monomer in a channel, forming a solid polymer that is extruded from the channel by fluid pressure. (*B*) Image of the E-SLIP process implemented in an annular geometry, with an inner black fluorinated ethylene propylene polymer tube (plugged) and outer glass channel shown. Images at different times during extrusion of a polymer tube. (*C*) Pressure over time during extrusion of monomer solution (without and with 20 wt.% PDMS-PEO) at a constant flow rate (Q = 1 mL/min), with time-averaged steady-state pressure shown as a red dotted line. (*D*) Steady-state pressure required to extrude an annular tube of photopolymer and thickness of lubrication layer formed with respect to varying weight fractions of PDMS-PEO block copolymer in the monomer solution, at a flow rate of 1 mL/min. (*E*) Representative tensile stress–strain curves of thiol-ene resins (containing 20 wt.% PDMS-PEO) used in extrusion, whose mechanical properties can be tuned from flexible to rigid by varying the molar ratio between acrylate components, PEGDA (polyethylene glycol diacrylate) and PETA (pentaerythritol tetraacrylate), with representative parts fabricated by E-SLIP shown in insets.

The method of synthetic tip growth described herein features the continuous, local photopolymerization of a monomer solution supplied by pressure-driven flow. While the light-based fabrication of microfibers (32, 35) or microparticles (34) via simultaneous flow and photopolymerization in microfluidic devices has been previously reported, there is no similarly available technique for simultaneous flow and photopolymerization on the macroscale that could enable synthetic tip growth. Thus, we established a methodology, broadly outlined in Fig. 2*A*, in which a flowing monomer solution constrained in a stationary transparent channel is selectively photopolymerized into a solid crosslinked network in an illuminated region. The cured polymer completely fills the channel and is expelled by the incoming monomer solution due to fluid pressure. In this work, a thiol-ene–based monomer chemistry was selected due to its rapid curing kinetics (35), low oxygen inhibition (36), low shrinkage (37), and commercial availability of monomer species.

The main challenge of this process lies in overcoming the frictional and adhesive forces that arise from channel–polymer interactions, such that continuous extrusion is possible. Adhesion is expected to arise mainly from chemical bonding and physical interlocking between polymer and the glass channel wall (38). A fluorinated silane release agent, previously shown to reduce adhesion and improve the release properties of glass (39), was applied to the glass surface of the channel to significantly reduce the surface energy of the glass (*SI Appendix*, Fig. S1). However, successful extrusion was not possible through this surface modification alone. Limiting adhesion is a crucial challenge in light-based additive manufacturing, which has been previously mitigated by the introduction of a nonreactive layer in between the resin and solid interface, either by polymerization inhibition (28) or use of an immiscible liquid (29). Building on this concept, we introduce a block copolymer amphiphile, poly(dimethylsiloxane)-graft-poly(ethylene oxide)-grafted copolymer (PDMS-PEO) containing ∼65% poly(ethylene oxide) content by weight, to the monomer solution as a lubricating component (see *SI Appendix*, section 1). By doing so, we successfully extruded solid photopolymerized polymer (Fig. 2 *B* and *C*). As discussed below, we believe that selective adsorption of the block copolymer onto the channel wall facilitates the formation of a liquid interface composed predominantly of PDMS-PEO (*SI Appendix*, Fig. S2). Due to the presence of the block copolymer in the bulk monomer solution, the lubricating layer forms spontaneously and is replenished continuously during extrusion. The incorporation of PDMS-PEO lubricant in the monomer solution is essential; when extruding monomer solution without lubricant but instead precoating the channel walls with lubricant, the lubricant layer is inevitably depleted and extrusion eventually fails. By having the monomer solution replenish the lubricant layer, continuous extrusion is possible; we call the developed fabrication methodology extrusion by self-lubricated interface photopolymerization (E-SLIP).

The E-SLIP process was implemented in an annular channel setup shown in Fig. 2*B* and Movie S1. Monomer solutions prepared with various concentrations of PDMS-PEO were extruded at a constant flow rate. The fluid pressure was measured simultaneously during extrusion (Fig. 2*C*) to probe adhesion and friction at the channel–polymer interface. The fluid pressure was a natural variable to monitor, due to the flow-rate–controlled nature of extrusion and the fact that fluid pressure is likely to be sensitive to changes in flow rate as well as friction between the channel and the polymer. The fluid pressure remains constant prior to ultraviolet (UV) light-emitting diode (LED) illumination. After illumination, the fluid pressure rapidly rises, likely due to initial static friction between the initially formed solid photopolymer and channel. In the case of extrusion with 20 wt.% PDMS-PEO in the monomer solution, the fluid pressure decreases to a steady-state value, as the cured photopolymer tube initially exits the channel, which is expected to coincide with a fully developed lubrication layer (thickness ∼30–45 µm) at the interface (*SI Appendix*, section 2 and Fig. S3). We note the fluid pressure fluctuates during steady-state extrusion, which is consistent with slip-stick phenomena. However, without PDMS-PEO in the monomer solution, no lubrication layer is formed and adhesion between the wall and polymer is significant enough to prevent extrusion, as the fluid pressure continues to rise until device failure.

By increasing the PDMS-PEO content in the monomer solution, the steady-state extrusion pressure was substantially reduced (Fig. 2*D*) and the inferred thickness (L_lubricant_) of the lubricating layer increased. With increased PDMS-PEO concentration, the coverage of the glass surface by the lubricant increased during the wetting process, thus leading to decreased extrusion pressure. While the mechanism for the formation of the lubrication layer is still under investigation, our working hypothesis is that lubrication is initiated by preferential wetting of the PDMS-PEO block polymer, due to its amphiphilic nature, as compared to the monomer solution without block copolymer (*SI Appendix*, Fig. S1). To understand the role that the channel surface plays in dictating whether continuous extrusion is possible, we conducted extrusion of the monomer solution with 20 wt.% PDMS-PEO with an untreated glass channel, which possesses a much larger surface energy (66 mN/m^2^) than the fluorinated channel. We found that continuous extrusion is not possible due to large slip-stick events that lead to ejection of the polymer tube from the channel as built-up pressure releases during a slip-stick event (*SI Appendix*, Fig. S4). The presumption is that the surface energy of the channel walls must play an important role in promoting localization of the block copolymer at the interface.

By utilizing a low-viscosity (∼50 mPa-s, *SI Appendix*, Fig. S5) liquid monomer and a self-lubricated interface, E-SLIP operates at low pressures (∼10 kPa) and requires only simple and inexpensive equipment. By confining solidification inside the channel and using low-shrinkage resins (*SI Appendix*, Fig. S6), excellent dimensional fidelity to the channel geometry was observed (*SI Appendix*, Fig. S7), with the channel geometry dictating the final shape. This allows the structure to be templated and then generated fully before exiting from the supporting channel. Moreover, by changing the geometry of the channel, a variety of profiled shapes can be accessed beyond the tube structure (*SI Appendix*, Fig. S8).

An inherent advantage of photopolymerization as the method for forming the solid structure is the ability to generate parts with a broad range of physical and mechanical properties by tuning the chemistry (40). E-SLIP is able to fabricate robust parts (Fig. 2*E*) with elastic modulus spanning two orders of magnitude (from 7 to 570 MPa, see *SI Appendix*, Table S1 for further details on mechanical properties), simply through modulation of the molar ratio of two acrylate components in the monomer solution. The addition of PDMS-PEO to the monomer solution results in some reduction in the stiffness of the solid resins due to plasticization of the network (*SI Appendix*, Fig. S9 and Table S1), with the elastic modulus reduced from 11 MPa to 7 MPa in the case of the flexible resin. We note E-SLIP is likely to be compatible with other photochemistries, expanding the design space of possible functional monomers and block copolymers.

**Fig. 3.**
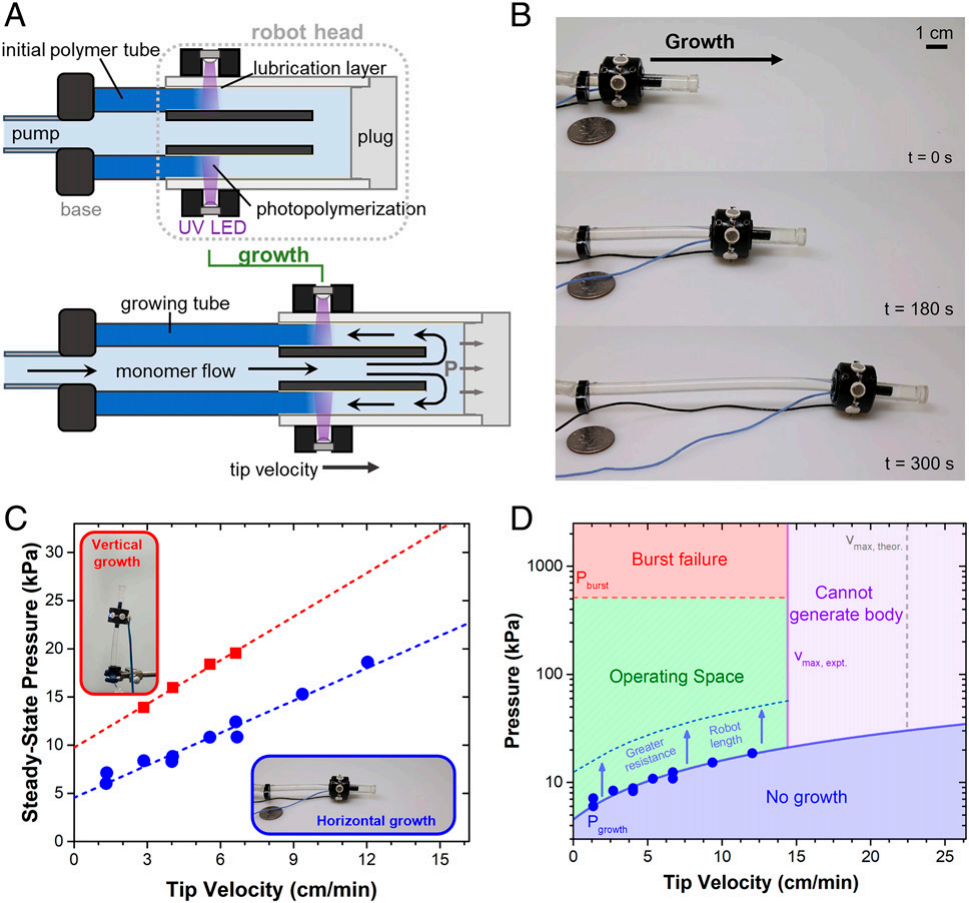
A soft robot that grows at the tip through E-SLIP. (*A*) Growth via photopolymerization at the tip. Constant flow of monomer supplied to the tip drives growth by moving the head forward and generating the robot body through continuous photopolymerization. (*B*) Images of growing robot over time, at a flow rate of 1 mL/min (Movie S2). (*C*) Pressure behavior as a function of tip velocity in two different growth directions, horizontal and vertical. Dotted lines are respective linear fits. (*D*) Operating diagram for a horizontal growing robot with bounds of a minimum pressure for growth, the maximum theoretical tip velocity determined from photopolymerization kinetics, and the upper pressure at which the photopolymerized tube bursts. Bounds determined by theoretical calculations are shown as dotted lines, with experimental determined bounds shown as solid lines.

E-SLIP, implemented in a growing soft robot (Fig. 3*A*), enables the three identified principles of biological tip growth to be realized in a synthetic growing system. The robot’s extension is initiated with a short length of polymer tube (∼5 cm), extruded previously using E-SLIP in the setup shown in Fig. 2*A*. This tube serves as the starting channel to deliver monomer solution to the robot head, paralleling the fluid-mediated transport principle in biological systems. In the robot head, the flow of monomer is directed back through an annular channel and past a set of circumferential UV LEDs for localized photopolymerization, analogous to the local cell wall synthesis. The fluid pressure propels the robot head forward, enabling the continuous construction of the robot body, which mirrors the turgor-pressure–based driving force in biological systems. Once formed, the photopolymerized body remains static relative to its surroundings, with only the robot head moving relative to the environment. Utilizing this bioinspired approach, our soft robot generates its own solid polymer structure and can lengthen by hundreds of percent (Fig. 3*B*). We have found that the ultimate length of the grown body has been limited only by the amount of monomer in the reservoir; however, the maximum growth lengths possible are likely dictated by the material properties of the selected monomer and polymer (see *SI Appendix*, section 7).

To elucidate the growing behavior of the robot, the fluid pressure was measured during robot lengthening on a white poly(tetrafluoroethylene) substrate with the monomer solution driven at a constant flow rate. This fluid pressure behavior resembles that observed in the extrusion setup, with an initial rise in pressure and then a decline to a steady-state value (*SI Appendix*, Fig. S10). This pressure increased linearly with the robot tip velocity, which is directly proportional to the liquid monomer flow rate (*SI Appendix*, Fig. S11 and section 3), with a minimum pressure extrapolated to zero velocity (Fig. 3*C*). We note that this positive linear velocity dependence of the extrusion pressure is characteristic of hydrodynamic friction (41), originating from the lubricant layer between the confining channel and solidified polymer. In other words, the bulk of the pressure to grow can be attributed to overcoming the friction at the interface, not the pressure drop due to fluid flow through the body and robot head (*SI Appendix*, section 4). The minimum pressure is attributed to the resistive forces that are overcome during growth: the internal friction between the polymerized tube and the channel walls and the external resistance between the robot head and the environment. The internal friction, as reflected in the steady-state pressure, is reduced with increasing PDMS-PEO concentration (*SI Appendix*, Fig. S12 and section 5), consistent with the extrusion case in Fig. 2*D*.

When switching the direction of growth from horizontal to vertical, an upward shift in the pressure–velocity relationship was observed, attributed to an increase in external resistance due to the weight of the robot head (∼15 g). Unsupported vertical growth is only possible for finite lengths of growth, before buckling of the robot body due to the flexible nature of the resin used (*SI Appendix*, Fig. S13). Note that, even though the tube buckles under this condition, the growth continues. To allow for continued growth in the vertical direction, growth of the robot was confined to a channel before the buckling point was reached. Increasing the stiffness of the resin would likely delay this buckling point to larger length scales.

The tip velocity and the fluid pressure are key parameters that govern the behavior of our soft robot. The growth behavior is constrained in pressure and velocity on three boundaries: a minimum steady-state pressure required to overcome the internal and external resistive forces, a maximum pressure dictated by the burst pressure of the robot body, and a maximum velocity determined by the photopolymerization kinetics of the monomer solution. We present these boundaries (Fig. 3*D*) for this specific robot growing unimpeded in a horizontal path with a specific set of operating parameters and other variables. Operating parameters and environmental conditions, such as light intensity, chemistry selected, and robot geometry, codetermine such bounds.

A large window of operating pressures is possible, as indicated by the difference between the burst pressure (*SI Appendix*, section 6) and growth pressures, which spans nearly two orders of magnitude. This allows the robot to extend in environments with higher impedance (e.g., media such as loose soil), which require a higher operating pressure. This also suggests that growth is only possible in solid photopolymers that form tubes with a burst pressure (which is directly proportional to tensile strength) that exceeds the pressure required for growth. Additionally, this pressure operating window can accommodate large extensions due to the small pressure gradient associated with transporting the low-viscosity monomer solution through the supply tube. Growth is possible provided the pressure required for growth does not exceed the burst pressure of the tube being formed. An upper bound for total length can be estimated, assuming increases in pressure to grow are solely derived from length increases and associated increases in pressure drop due to fluid flow. Using this approach, a theoretical maximum grown length of 3,800 m was estimated for the flexible resin used in this work (see *SI Appendix*, section 7). This maximum length is a function of several material parameters, such as tensile strength and viscosity, as well as tube geometry.

The maximum steady-state tip velocity is strongly dependent on the photopolymerization kinetics. Successful growth requires rapid photopolymerization that solidifies the monomer solution before it exits the illuminated region. This implies a maximum tip velocity $\left(v_{\max}\right)$ given by the following expression:

[1] $$v_{\max}=\frac{d_{\mathrm{light}}}{t_{\mathrm{gel}}},$$

where *d_light_* is the length of the illuminated region along the direction of flow and *t_gel_* is the timescale required for the liquid–solid transition to occur (gel time) throughout the full thickness of the annular channel. Due to attenuation of light intensity in the monomer solution, the gel time varies radially, with monomer furthest from the light source exhibiting the highest gel time and thus limiting the timescale for growth. Therefore, the maximum tip velocity is also dependent on thickness of the channel used, with thicker channels having a reduced maximum tip velocity. Gel times were determined from Fourier transform infrared spectroscopy kinetic experiments with a model to account for light attenuation (*SI Appendix*, Fig. S14 and section 8). The upper velocity limit was also determined experimentally by linearly increasing the flow rate and associated robot velocity until device failure due to incomplete photopolymerization (*SI Appendix*, Fig. S15). The discrepancy between the theoretical and experimental upper velocity limit likely arises from the assumptions of this model. One assumption is that absorption is unchanged during photopolymerization; however, an increase in light attenuation after photopolymerization was observed (*SI Appendix*, section 9 and Fig. S16). We also assume that the mechanical properties at the liquid–solid transition (gel point) are robust enough to support the applied pressure, which is likely an overestimation.

**Fig. 4.**
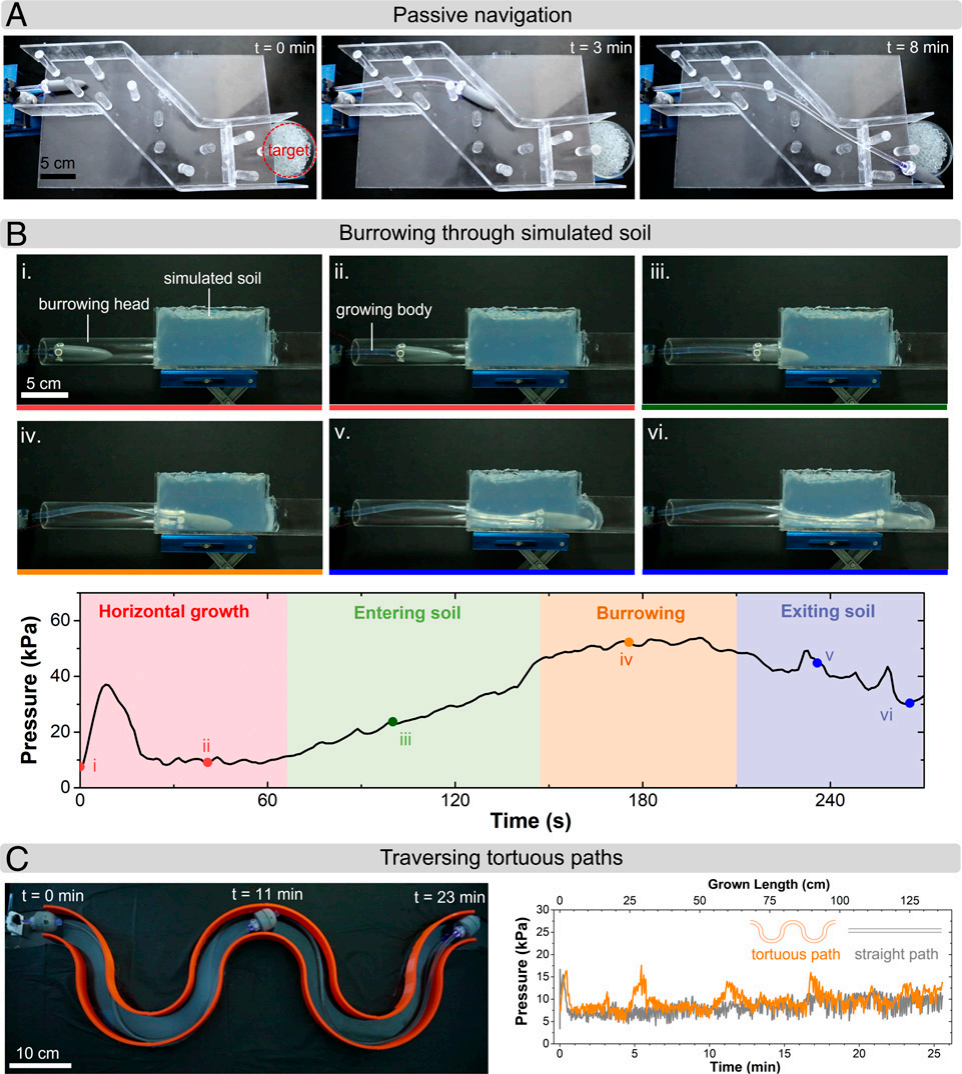
Growth of soft robot in constrained environments and tortuous paths. (*A*) Due to the compliance of the growing robot, it is able to deflect and bend around obstacles as it grows in a constrained path toward a target (Movie S3). Each panel represents a different time snapshot during growth. (*B*) Burrowing of the growing robot through a transparent soil analog. The different panels represent different times during growth and are indexed to the pressure–time plot by Roman numerals (i–vi). Fluid pressure stays at steady state before entry into soil, with increases observed before reaching a new steady state while burrowing due to the higher impedance of the soil substrate. Pressure then falls off as the robot exits the soil (Movie S4). (*C*) Overlayed images showing growth through a tortuous path, with time stamps to show time elapsed. By utilizing a liquid-mediated transport of feed materials for the growing tip, the robot is capable of taking tortuous paths (Movie S5) with pressure increases with length that are comparable to those of straight growth. Pressure spikes during growth along the tortuous path coincide with robot head reorientation during turning.

Our soft robot demonstrates many of the capabilities of biological tip growth, in particular, passively navigating its environment, traversal of tortuous pathways, and movement in constrained environments. The robot, equipped with a nose cone, can passively steer around obstacles and reach a desired destination along a constrained path (Fig. 4*A* and Movie S3). This capacity arises from the robot’s inherent compliance, which permits it to conform to its environment and follow the path of least resistance. We also investigated the ability of the robot (modified for burrowing, *SI Appendix*, Fig. S17) to grow through higher-impedance environments, i.e., a transparent soil analog (*SI Appendix*, Fig. S18). The robot exhibited a root-like ability to burrow through an impeded path while simultaneously generating a 3D structure (Fig. 4*B* and Movie S4). Variations in fluid pressure were observed to correspond with the robot’s interactions with the environment: a steady-state pressure for unimpeded horizontal growth, a rising pressure while entering the simulated soil, a constant burrowing pressure with a fully submerged head, and a declining pressure exiting the simulated soil. At a known flow rate, sensing pressure deviations at the base due to obstacles or changing environments encountered at the growing tip provides a method to sense basic tip–environment interaction, resembling plant root stimuli response to changing soil mechanical impedance. Such sensing may be used to inform robot growth or to help map the environment through which it is traveling.

Living organisms routinely exhibit the ability to navigate long, tortuous pathways; this ability has been fundamentally stymied in a man-made tip-growing system lacking fluid-mediated material transport. Previously developed growing robots drag solid-state supply lines along with them as feed for new growth. As such robots grow along tortuous paths, the force needed to drag these solid tethers increases exponentially (42). These limiting forces arise due to the friction between the solid material supply and the generated walls, which scales with moving contact area following “capstan equation” behavior (42). By emulating the liquid-mediated transport principle seen in nature, our growing robot can forgo the solid tether (with the use of a battery-powered head, *SI Appendix*, Fig. S19) in favor of a liquid material supply circumventing this dominant source of friction. When growing our robot around a tortuous path (Movie S5), there is a negligible change in the operating pressure as a function of the robot length (with a final length of ∼1.5 m), as compared with a straight-line path (Fig. 4*C*). This suggests that the tortuosity of the path has limited (subexponential) effect on the growing behavior, opening the possibility of the traversal of paths with significant tortuosity.

## Conclusions

We have demonstrated a synthetic method of growth, E-SLIP, inspired by biological principles used by fungal hyphae, plant roots, and pollen tubes. E-SLIP allows the production of profiled polymer parts directly from monomer solution through photopolymerization within a channel. A growing soft robot, utilizing E-SLIP, was capable of lengthening many times its original body length, burrowing in a simulated soil, passively avoiding obstacles, and traversing tortuous paths. By employing different chemistries and robot designs, a greater range of functionalities, such as biodegradability, improved mechanical properties, and active steering, could be accessed. Our approach offers the possibility of a new materials-processing and growing robot platform for on-demand infrastructure, exploring, and sensing in a variety of confined, remote, or hard-to-access environments.

## Materials and Methods

### Monomer solution preparation.

All chemicals were purchased from Sigma-Aldrich, unless otherwise noted, and used as received. The thiol-ene–based monomer solution consisted of a multifunctional acrylate component, either poly(ethylene glycol) diacrylate (PEGDA) (molecular weight = 700 g/mol for flexible resin) or pentaerythritol tetraacrylate (PETA) (TCI America for rigid resin), or blend thereof, combined with the tetra-functional thiol (pentaerythritol tetrakis(3-mercaptopropionate) in a molar ratio of 8:5 acrylate to thiol functional groups. These monomers were combined in a scintillation vial with 0.15 wt.% photoinitiator (diphenyl(2,4,6-trimethylbenzoyl)phosphine oxide), and a radical scavenger, propyl gallate, was added at 0.1–0.2 wt.% to act as a stabilizer, preventing premature polymerization of the thiol-ene (43). To the monomer solution, 20 wt.% (unless specified otherwise) of the lubricating component, poly(dimethylsiloxane)-poly(ethylene oxide) graft copolymer (Gelest, DBE-712) was added and stirred for several minutes until completely dissolved. For use in extrusion or the growing robotic device experiments, the monomer solution was transferred to a Luer lock syringe.

### Glass channel surface treatment.

Protocol for surface treatment of glass channels followed previous literature procedures (39). Briefly, quartz and borosilicate glass tubes (McMaster-Carr) were first treated in a base bath (isopropyl alcohol, potassium hydroxide, and distilled water) for several hours to eliminate organic contamination, rinsed in distilled water, and then plasma treated in a Harrick Plasma cleaner (PDC-32G) for 5 min. The plasma-treated glass was then added to a 2 wt.% solution of tridecafluoro-1,1,2,2-tetrahydrooctyl dimethylchlorosilane (Gelest) in toluene to generate a fluorinated self-assembled monolayer and left in the solution for at least 24 h and then rinsed successively with isopropyl alcohol and distilled water and heated in an oven for 2 h at 120 °C before use. All extrusion and growing robot experiments utilized these fluorinated glass channels.

### Annular extrusion setup and hardware.

The E-SLIP setup consisted of the following main elements: a programmable syringe pump (Harvard Apparatus PhD Ultra), connective tubing (Tygon, outer diameter = 3/16 in), and transparent channel with light source for photopolymerization. The channel was made up of the fluorinated quartz tube, containing within it an annular region, composed of a UV-blocking fluorinated ethylene propylene (FEP) (McMaster-Carr) tube held in place concentrically with an additively manufactured custom holder (Clear Resin on Formlabs 1+). During extrusion, this FEP tube is plugged to prevent flow within. The light source was composed of six concentric UV-light–emitting diodes (Chanzon, λ = 380 nm, 3 W, 6-mm lens diameter), which surrounded the surface-treated quartz tube and were masked to allow a 5-mm window of light (*d_light_*) to reach the glass tube through an additively manufactured housing (made with PLA on a MakerBot Replicator 2). Flow rate was set and recorded through the status monitor on the syringe pump, and pressure data were monitored through an electronic pressure sensor (0–206.84 kPa [30 psi] Honeywell TBP series, vented gauge) positioned at a three-way tubing junction near the monomer injection port at the robot base. Silicone oil was used as an immiscible buffer fluid between the monomer solution and pressure sensor to prevent premature sensor failure. These sensors were connected to a custom data acquisition circuit, with a microcontroller (Teensy 4.0) coded to control and record the cameras, UV LEDs, and sensors. To conduct E-SLIP, a Luer-lock syringe was loaded on the syringe pump and mated with the connective tubing. Monomer was initially supplied to fill the channel, oriented vertically, up to the point of the UV LEDs. Extrusion was initiated by starting flow of monomer (at a constant flow rate) through the syringe pump and simultaneously supplying power to the UV LEDs.

### Growing robot setup and hardware.

The growing robot process utilized the same hardware used in extrusion. The robot head consisted of the glass channel with an unplugged FEP inner tube, UV LEDs and housing, and an additively manufactured end plug (made with Flexible Resin on Formlabs 1+). A short length (∼5 cm) of previously extruded photopolymer tube was used as the connection between the robot head and the connective tubing extending from the syringe, held in place with plastic snap-grip clamps and a tapered plastic tubing connector. The polymer tube and robot head were filled with monomer in a vertical orientation to eliminate air bubbles from the tube and channel. The end plug was inserted in the channel to fully seal the channel. Growth could then be started by the simultaneous flow of monomer from the syringe and powering of UV LEDs.

## Supplementary Material

Supplementary File

Supplementary File

Supplementary File

Supplementary File

Supplementary File

Supplementary File

## Data Availability

All data needed to evaluate the conclusions in the paper are present in the main text and the *SI Appendix*. All study data are included in the article and/or supporting information.

## Change History

August 12, 2022: Due to a production error, the SI movies appeared incorrectly. The SI movies have been updated.
