# Supplementary material for: Synthetic growth by self-lubricated photopolymerization and extrusion inspired by plants and fungi
Source: Proc Natl Acad Sci U S A. 2022 Aug 9;119(33):e2201776119. doi: 10.1073/pnas.2201776119 (PMC9388119; doi:10.1073/pnas.2201776119)
Supplement: Supplementary File [file pnas.2201776119.sapp.pdf]

PNAS
www.pnas.org

Supplementary Information for

Synthetic growth by self-lubricated photopolymerization and extrusion inspired by plants and fungi

Matthew M. Hausladen[1], Boran Zhao[1], Matthew S. Kubala[2], Lorraine F. Francis[1], Timothy M. Kowalewski[2*], and Christopher J. Ellison[1*]

[1]Department of Chemical Engineering and Materials Science, University of Minnesota, Minneapolis, MN 55455, United States.

[2]Department of Mechanical Engineering, University of Minnesota, Minneapolis, MN 55455, United States.

*Corresponding authors: cellison@umn.edu (C. J. Ellison); timk@umn.edu (T. M. Kowalewski)

This PDF file includes:

Materials and methods
Supplementary text
Figures S1 to S19
Tables S1 to S2
Legends for Movies S1 to S5
References

Other supplementary materials for this manuscript include the following:

Movies S1 to S5

Materials and methods

Materials. Poly(ethylene glycol) diacrylate (PEGDA, molecular weight = 700 g/mol), pentaerythritol tetrakis(3-mercaptopropionate (PETMP), diphenyl(2,4,6-trimethylbenzoyl)phosphine oxide (TPO), propyl gallate, silicone oil, and mineral oil were purchased from Sigma Aldrich and used as received. Pentaerythritol tetraacrylate (PETA) was purchased from TCI America and used as received. Poly(dimethylsiloxane)-poly(ethylene oxide) graft copolymers with varying molecular weights and poly(ethylene oxide) content (DBE-224, DBE-311, DBE-411, DBE-621, and DBE-712, see Table S2) and tridecafluoro-1,1,2,2-tetrahydrooctyl dimethylchlorosilane (DMCS) were purchased from Gelest, and used as received. Hydrophobic fumed silica (Aerosil R812) was purchased from Evonik. Quartz glass tube (outer diameter = 9mm, inner diameter = 7mm) and fluorinated ethylene propylene (FEP) polymer tubing (outer diameter = 4mm, inner diameter = 2mm) were purchased from McMaster-Carr.

Tensile testing. For mechanical testing, monomer solution was placed in Teflon molds shaped according to ASTM standard D1708 and exposed to ultraviolet (UV) light for 10 minutes at 20 mW/cm^2. Uniaxial tensile tests were conducted on a tensile tester (Instron 5966), equipped with a 500N load cell at a strain rate of 1 mm/min. The mechanical properties reported were the averages of at least three specimens.

Fourier-transform infrared (FT-IR) spectroscopy. FT-IR spectroscopy was conducted with a Nicolet 6700 FTIR spectrometer (Thermo Fisher Scientific) with a KBr beam splitter, mercury cadmium telluride-A (MCT-A) detector. FT-IR spectra of liquid samples (PDMS-PEO, monomer solution, etc.) were obtained using an attenuated total reflectance (ATR) fixture. An FT-IR spectrum of the lubricating layer was taken by wetting the ATR crystal with an extruded polymer tube with excess lubricating layer fluid originating from the tube exterior. The kinetics of the thiol-ene photopolymerization for the thiol-ene monomer solution were characterized by real-time FTIR scans using a customized horizontal transmission accessory, with UV irradiation generated using a mercury lamp UV light source (OmniCure S1500 Spot UV Light Curing System; Excelitas Technologies). Optically thin samples (~20 µm) were prepared by placing monomer solution between two polished NaCl plates. Acrylate conversion ($C_{acrylate}$) was tracked by monitoring peak area change for the C=C peak (1630 cm^{-1}), normalized by the C=O peak (1702 cm^{-1}), which does not participate in the polymerization, which is shown in Eq. S1.

$$C_{acrylate} = 1 - \frac{A_{acrylate}(t)}{A_{acrylate}(t=0)} \qquad \text{(Eq. S1)}$$

Photopolymerization kinetics were assessed at several UV intensities (5, 10, 15, 30, and 100 mW/cm^2), which were confirmed by a UV radiometer (Coherent FieldMaxII).

Surface energy and contact angle measurements. Contact angle measurements were conducted on a Kruss DSA305 goniometer system. The static contact angle was measured to calculate the surface energy of materials in the tip growing setup with the two-liquid method using water and methylene iodide following Fowkes theory as shown in Eq. S2 and S3.

$$\sqrt{\gamma_{l1}^{d}\gamma_{s}^{d}} + \sqrt{\gamma_{l1}^{p}\gamma_{s}^{p}} = \frac{\gamma_{l1}(1+\cos\theta_1)}{2} \qquad \text{(Eq. S2)}$$

$$\sqrt{\gamma_{l2}^{d}\gamma_{s}^{d}} + \sqrt{\gamma_{l2}^{p}\gamma_{s}^{p}} = \frac{\gamma_{l2}(1+\cos\theta_2)}{2} \qquad \text{(Eq. S3)}$$

where γ is either the surface tension or energy of materials, subscript *l1*, *l2* and *s* denote the two testing liquids used (1 for water, 2 for methylene iodide) and the surface of interest, the superscript *d* and *p* stand for the disperse and polar component of the surface tension/energy of materials, and finally θ_1 and θ_2 are the static contact angle of water and methylene iodide on the surface of interest. The results of surface energy and contact angle are summarized in Figure S1.

Lubricating layer measurement. Indirect measurement of the lubricating layer was conducted by measurement of the photopolymer tube diameter after extrusion using a DHR-3 (TA Instruments) rheometer with a 25 mm parallel plate geometry, due to the extensible nature of the photopolymer tubes. Plates were lowered until a non-zero axial force (>0.2g) was measured, with the gap at this point taken as the outer diameter of extruded tube. The approximate average lubrication layer thickness ($L_{lubricant}$) was then inferred from the extruded tube outer diameter (d_{tube}) and the glass channel inner diameter ($d_{glass,\ inner}$), by the following equation, which assumed a uniform lubrication layer:

$$L_{lubricant} = \frac{d_{glass,inner} - d_{tube}}{2} \qquad \text{(Eq. S4)}$$

Preparation of transparent simulated soil. The transparent simulated soil was prepared by mixing 15 wt.% of hydrophobic fumed silica in mineral oil and then mixed using a motorized mixer, according to previous work (1). The synthetic soil was further dispersed and degassed for five minutes using a planetary mixer (Thinky ARE-310). Upon addition of the simulated soil to the burrowing testbed, the setup was placed under vacuum to remove any remaining air.

Rheology. All rheology experiments were conducted with a rotational rheometer (DHR-3, TA Instruments) with appropriate accessory and geometry. Shear rheology of the simulated soil was conducted using with a 25 mm parallel plate geometry, with a Peltier plate held at 25°C. Oscillatory amplitude sweeps were conducted at 1 Hz from 0.1-500 Pa in oscillatory stress. Shear rheology of the monomer solution containing 20 wt.% PDMS-PEO was conducted with a 40 mm cone and plate geometry, with a Peltier plate held at 25°C. Shear rate flow sweeps were performed from (0.1 to 100 Hz). Photorheology of the thiol-ene resins was conducted using a 20 mm upper plate and a transparent quartz lower plate to expose the sample to UV light. Samples were held at an initial gap of 300 um and the oscillatory time sweeps conducted at 1 Hz and 0.01% strain (in the linear regime). UV light was initiated at 20 seconds into the time sweep. Axial force was maintained at 0 N during photopolymerization and the gap was allowed to change to accommodate shrinkage. The linear shrinkage of the resin was estimated using changing gap using the following equation:

$$Linear\ shrinkage = \frac{Gap(0) - Gap(t)}{Gap(0)} \qquad \text{(Eq. S5)}$$

Extrusion temperature. Measurements of temperature of the extruded tube were taken after the part exited the channel during extrusion of the flexible resin using a thermocouple-based

thermometer (Thermo-Fischer). The maximum temperature was recorded and was found to be 68° C.

Supplementary text

Supplementary Section 1: Surface energy and lubrication design

The surface energies of surfaces in contact have a large effect on chemical adhesion experienced. Thus the surface energy of the glass channel walls was reduced substantially (Figure S1A) by treating with a fluorinated silane (DMCS), through generation of a fluorinated self-assembled monolayer on the glass surface. However, thiol-ene monomers photopolymerized in a fluorinated channel still suffered from substantial adhesion, likely due to mechanical adhesion effects. A non-reactive lubricating constituent was added to the monomer resin to introduce a liquid lubricating layer at the interface in an attempt to eliminate solid-solid contacts and any chemical bonding. To achieve this, the liquid lubricant should be miscible with the monomer resin (to allow for light penetration through the sample during extrusion) and preferentially adsorb at the interface. Several potential lubrication additives were screened (Table S2), with a PDMS-PEO block copolymer (with 65 wt.% PEO) selected as the lubricant additive for extrusion, due to its substantial miscibility with the monomer resin. The ability of the PDMS-PEO block copolymer to accumulate at the interface is in part due to its enhanced wetting over the monomer resin (Figure S1B), with a lower contact angle than the monomer resin on untreated and treated glass. Notably, this accumulation at the interface continues after extrusion of the part, with PDMS-PEO migrating to the polymer-air interface and generating a thin liquid film on extruded parts.

Supplementary Section 2: Pressure behavior as a function of time in extrusion

The fluid pressure was measured as a function of time during extrusion. Typical pressure-time curves during the E-SLIP extrusion in a ‘dry’ (unwetted with PDMS-PEO) channel are displayed in Figure S3A. The pressure first rises linearly at the beginning of extrusion, then reduces to a much lower but constant value with small fluctuations. The initial increase in pressure is partly attributed to the static friction between the newly formed tube and channel. Additionally, we observed that the pressure still rose despite the movement of the initially formed tube and that the time when the pressure value peaked typically coincided with the moment the initially formed section of tube first exited the channel. These observations indicate that the duration and magnitude of the initial rise in pressure is related to the ‘dry’ length of the channel, i.e., the unlubricated distance between the UV illumination window and the end of the channel prior to the E-SLIP extrusion, as illustrated in Figure S3B. The dynamic friction between the moving tube and channel is directly related to the establishment of a wetting layer, hence the longer the ‘dry’ distance, the higher the peak pressure value. This is confirmed by the pressure versus time plot from an extrusion with a pre-wetted channel from a previously run extrusion, which demonstrated both a lower peak pressure value and a shorter peak time (Figure S3A). Once a wetting layer is established, the extrusion pressure reduces and fluctuates about a constant value.

Supplementary Section 3: Growing robot velocity validation

To verify that the flow rates imposed by the syringe pump were being realized in the growing robot, a comparison between the expected velocity and the average experimental velocities was

conducted. The theoretical average growing robot velocity is determined by the following equation:

$$v_{robot,avg} = \frac{Q}{A_{total}} \qquad \text{(Eq. S6)}$$

where Q is the volumetric flow rate imposed by the syringe pump and A_{total} is the total cross-sectional area of the tube, which includes both the annular region of the photopolymer tube and inner monomer fluid region. This cross-sectional area determines robot velocity because for every new volume of photopolymer tube that is generated, there must be a matching volume of monomer fluid within the interior of the tube to ensure fluid continuity within the device.

The experimental average velocities were determined by manually measuring the length of photopolymer tube generated and dividing it by the total experimental time. This results in the average robot velocity for the entire experiment. The comparison between these experimental and theoretical robot velocities across several imposed flow rates (Figure S11), showing approximate agreement between the two.

Supplementary Section 4: Pressure drop due to monomer flow in growing robot

During growth, there are contributions to the pressure required to grow from friction and from the pressure required to transport monomer. To ascertain which contribution dominates, the pressure required to transport monomer from the syringe to the growing tip was estimated. This can be approximated by assuming the pressure drop due to transport can be treated as the sum of the pressures to transport in the three distinct regions: robot body, inner supply tube of the robot head, and annular region of the robot head, using Poiseuille flow of a Newtonian liquid in a pipe and annulus (2), respectively. This is summarized as follows:

$$P_{transport} = P_{body} + P_{inner\ tube} + P_{annulus} \qquad \text{(Eq. S7)}$$

$$P_{transport} = \frac{8\eta L_{body}Q}{\pi R_1{}^4} + \frac{8\eta L_{tube}Q}{\pi R_2{}^4} + \frac{8\eta L_{tube}Q}{\pi(R_3{}^4 - R_1{}^4 - \frac{R_3{}^2 - R_1{}^2}{\ln\left(\frac{R_3}{R_1}\right)})} \qquad \text{(Eq. S8)}$$

Where η is the monomer solution viscosity, L_{body} is the robot length, Q is the volumetric flow rate, and R_1 is the robot body inner radius, R_2 is the F supply tube inner radius, and R_3 is glass channel outer radius.

As an example calculation, the largest $P_{transport}$ to be expected during growth experiments would have the following parameter values, (Q = 4.5 mL/min, R_1 = 2 mm, R_2 = 1 mm, R_3 = 3.45 mm, η = 0.05 Pa·s, L_{body} = 30 cm, L_{ube} = 3 cm), and is calculated to be 0.49 kPa, which is nearly two orders of magnitude smaller than the pressure to grow at the same flow rate. Thus both the velocity-dependence and magnitude of pressure can be attributed to friction during growth.

Supplementary Section 5: Effect of PDMS-PEO in lubricating the growing robot

The effect the concentration of the PDMS-PEO block polymer on the pressures required for growth using the flexible thiol-ene resin was investigated to see the effect of lubrication on the lengthening of the growing robot. A qualitatively similar trend is seen in the growing robot (Figure S10) as compared to lubrication with PDMS-PEO in E-SLIP in the extrusion setup (Figure 2D), with substantial early reductions in the pressure required for growth with increasing PDMS-PEO content.

Supplementary Section 6: Burst pressure analysis

The maximum pressure that the system can handle was calculated as the burst pressure of the extruded tube. To accomplish this, hoop and radial stresses were calculated using Lamé's equations for thick-walled cylinders (Eq. S9). Along with these, an additional compressive stress was included from the fluid pressure on the forward end of the extruded tube (Eq. S10). Based on the stress-strain curves of the two explored photopolymer chemistries (Figure 2E), the flexible composition was treated as a ductile material and the rigid composition was treated as a brittle material based on the criteria that a failure strain under 5% is brittle and above 5% is ductile. To determine the maximum allowable pressure for the flexible chemistry, the stresses were combined using von Mises theory. This was then set equal to the flexible polymer's yield stress and the internal pressure was solved for (Eq. S11). To determine the maximum allowable internal pressure for the rigid photopolymer, modified Mohr's failure theory was used with the hoop stress being the dominant stress component. This was then set equal to the rigid chemistry's yield stress and the internal pressure was solved for (Eq. S12).

$$\sigma_{hoop} = \frac{{r_i}^2 P_i - {r_o}^2 P_o}{{r_o}^2 - {r_i}^2} + \frac{(P_i - P_o){r_i}^2 {r_o}^2}{({r_o}^2 - {r_i}^2) r^2},\ \sigma_{radial} = \frac{{r_i}^2 P_i - {r_o}^2 P_o}{{r_o}^2 - {r_i}^2} - \frac{(P_i - P_o){r_i}^2 {r_o}^2}{({r_o}^2 - {r_i}^2) r^2} \qquad \text{(Eq. S9)}$$

$$\sigma_{axial} = -P_i \quad \text{(Eq. S10)}$$

$$\sigma_{vm} = \frac{1}{\sqrt{2}}\sqrt{(\sigma_{hoop} - \sigma_{radial})^2 + (\sigma_{radial} - \sigma_{axial})^2 + (\sigma_{axial} - \sigma_{hoop})^2},\ \sigma_{vm} \geq \sigma_y \qquad \text{(Eq. S11)}$$

$$\sigma_{hoop} = S_{u,t} \quad \text{(Eq. S12)}$$

Here σ_{hoop}, σ_{radial}, and σ_{axial}, are the hoop, radial, and axial stresses respectively. P_i and r_i are the internal pressure and inner radius, P_o and r_o are the external pressure and outer radius, and r is the radial position within the photopolymer tube where the stress is calculated. In the above equations, $\sigma_{vm}$is the von Mises stress, σ_y is the yield stress of the flexible chemistry and $S_{u,t}$is the ultimate tensile stress of the rigid chemistry. The dimensions used in this calculation do not include the lubrication layer thickness which accounts for ~1% of the tube radius.

Supplementary Section 7: Maximum robot length analysis

As the tip of the unimpeded robot grows at a constant speed (implying constant pressure at the tip), there would be increases in pressure at the base (where monomer solution is supplied) over time due to the frictional losses of pumping a viscous fluid in a pipe of increasing length. Assuming all pressure increases after reaching steady-state growth stem from these frictional losses, a determination of the resulting maximum grown length of the robot can be made, which would occur when the operating pressure to grow reaches the burst pressure of the polymer tube near the base. This is done by isolating the two pressure components are required for robot locomotion: the pressure required for growth at the tip (P_{growth}) and the pressure to transport monomer from the base of the robot along its body to the growing tip ($P_{transport}$), and setting them equal to the burst pressure:

$$P_{burst} = P_{growth} + P_{transport} \qquad \text{(Eq. S13)}$$

The fluid pressure can be approximated by Poiseuille flow of a Newtonian liquid in a pipe, which is governed by the following equation:

$$P_{transport} = \frac{8\eta LQ}{\pi R^4} \qquad \text{(Eq. S14)}$$

where η is the monomer solution viscosity, L is the pipe length, Q is the volumetric flow rate, and R is the pipe inner radius. The pressure gradient, or additional pressure for transport required per unit length, is found by taking the derivate of Eq. S14 with respect to length:

$$\frac{dP}{dL} = \frac{8\eta Q}{\pi R^4} \qquad \text{(Eq. S15)}$$

By combining eq. S13 and S14, and solving for length, an expression for the maximum length dictated by Poiseuille flow can be obtained:

$$L_{max,Poiseuille} = \frac{(P_{burst} - P_{growth})\pi R^4}{8\eta Q} = (P_{burst} - P_{growth})\left(\frac{dP}{dL}\right)^{-1} \qquad \text{(Eq. S16)}$$

As an example, using a flow rate used in this work (Q = 1 mL/min), the dimensions of the generated tube (R = 2mm), viscosity of the monomer solution (0.05 Pa·s), and the P_{burst} (512 kPa) and P_{growth} (8 kPa) for the PEGDA-based monomer resin yields a theoretical L_{max} of 3,800 m.

Supplementary Section 8: Photopolymerization kinetics and maximum velocity modeling

The growing mechanism requires that a complete annular cross-section become photopolymerized by the end of the illuminated region (Fig 2A, violet band) to prevent leakage of monomer. Some deviation along the inner or outer annular radii may be tolerated, resulting in a lower conversion and reduced mechanical properties. However, any deviation such that a part of the annular cross-section is not solidified would result in leakage that could catastrophically disrupt further extrusion or growth. Assuming a constant velocity profile, all monomers are exposed to UV light for the same amount of time. The timescale of photopolymerization can be captured in the gel time, which is the time required to reach the point of the liquid-solid transition. Past the gel point, the polymer network would be able to bear load and prevent monomer leakage. Thus, if the residence time of monomer in the illuminated region is less than the gel time, photopolymerization will not occur. However, gel times are not the same across the annular channel due to light attenuation through the channel width. The limiting photopolymerization timescale of interest for robotic device operation is the gel time at the channel wall opposing the light source. Given these conditions, the maximum velocity achievable by the growing robot before failure is:

$$v_{max} = \frac{d_{light}}{t_{gel}} \qquad \text{(Eq. S17)}$$

where d_{light} is the lengthscale of the illuminated region (Fig 2A, violet band) and t_{gel} is the gel time of the monomer fluid at the channel side farthest from the UV light source. To determine the gel time as a function of channel depth, the simulated UV intensity decay as a function of depth was calculated according to a Beer-Lambert relation:

$$I(z) = I_0 \, (10^{-\epsilon[PI]r}) \qquad \text{(Eq. S18)}$$

Where I_0 is the incident light intensity at the channel wall nearest to the light source, ε is molar absorptivity of the photoinitiator, [PI] is the concentration of photoinitiator, and r is the radial position from the light source. The molar absorptivity for the photoinitiator TPO was determined to be 60 m^2/mol and the initial intensity of the UV LEDs determined by radiometer to be approximately 12 mW/cm^2. Light attenuation based on the introduced version Beer-Lambert law assumes that the photoinitiator is the main source of light absorption and that the light absorption does not change much with time.

To ascertain the gel time as a function of channel depth, the photopolymerization kinetics were characterized via real-time FTIR to generate conversion curves of the thiol and acrylate group during photopolymerization at several different UV intensities. Thiol-acrylate polymerizations are known to undergo mixed chain and step growth polymerizations and Eq. S19 developed by Reddy and coworkers (3) allows for the calculation of the gel point in terms of acrylate conversion:

$$\frac{2}{r}\left(f_{acrylate} - 1\right)\frac{k_{CC}}{k_{CS}} p_\alpha + \left(f_{acrylate} - 1\right)\left(f_{thiol} - 1\right)\left(1 + \frac{2}{r}\frac{k_{CC}}{k_{CS}}\right) {p_\alpha}^2 = 1 \qquad \text{(Eq. S19)}$$

where r is the stoichiometric ratio between S-H and C=C functional groups, f_{acrylate} and f_{thiol} are the acrylate and thiol monomer functionalities, respectively, and k_{CC}/k_{CS} is the ratio of the propagation constant for the acrylate homopolymerization to the chain transfer constant for the thiol–acrylate reaction. Previous literature has demonstrated that k_{CC}/k_{CS}=1.5 for thiol-acrylate photopolymerization (4). The conversion at the gel point was determined to be 0.06 and 0.16 for the flexible and rigid thiol-acrylate resins, respectively. Gel times for each resin were determined for each different UV intensity, by a linear fit in the low conversion regime to find both the initial time at zero conversion and time at gel point, with the gel time the difference of the two, (Figure S14A, B). The films used in the FTIR study were approximately 20 μm in thickness and were treated as an adequate approximation for photopolymerization kinetics at zero thickness, due to negligible light intensity differences throughout the thickness.

It has been previously demonstrated that the gel times should scale with $I^{-½}$ in a thiol-acrylate photopolymerization (5). Gel times were plotted as a function of $I^{-½}$, shown in Figure S14C, yielding approximate linear fits and a resulting empirical relationship between gel time and UV intensity. Combining Eq. S18 with the linear fits yielded a relationship between depth and gel time for each distinct resin (Figure S14D), which allows for determination of t_{gel} and therefore v_{max}, which were calculated to be 22.2 cm/min and 154 cm/min for the flexible and rigid resins, respectively.

Supplementary Section 9: Light absorption in monomer solution and polymerized film

In the maximum velocity model, there is an assumption that absorption does not vary substantially during photopolymerization and the photoinitiator is the main source of absorption. However, while these assumptions are not rigorously met, as there is increased light attenuation in the photopolymerized film (Figure S16), the model does appear to capture dominant variables that influence photopolymerization and their scaling.

Supplementary Figures

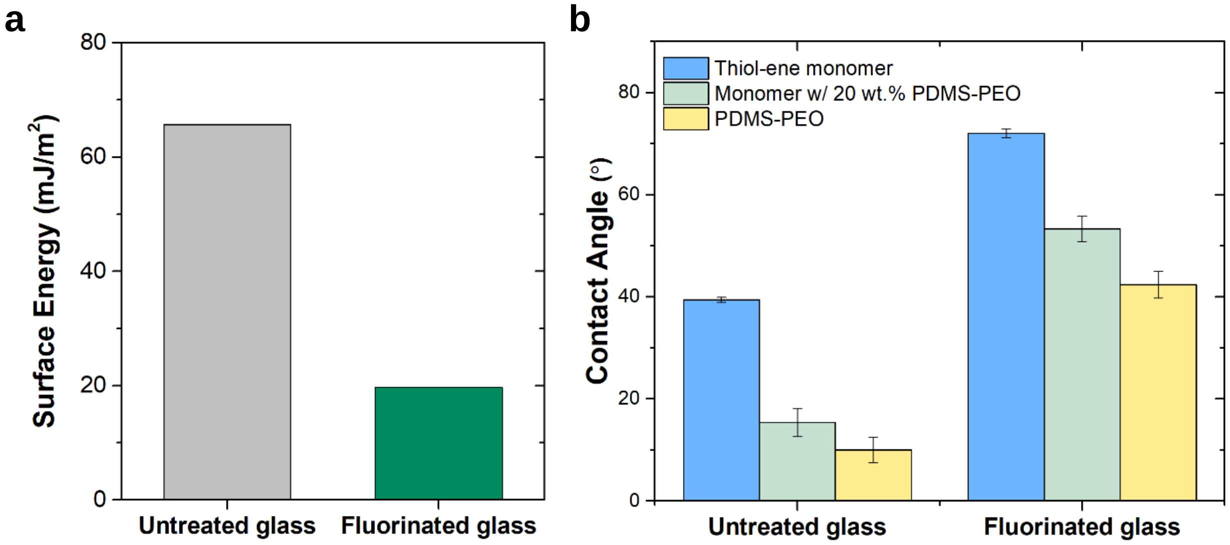

Figure S1. Surface energy and contact angle measurements. (a) Surface energy of glass before and after silanization surface treatment with DMCS. **(b)** Contact angle measurements of thiol-ene monomer resin without PDMS-PEO, monomer solution containing 20 wt.% PDMS-PEO, and PDMS-PEO block polymer on untreated glass and fluorinated glass.

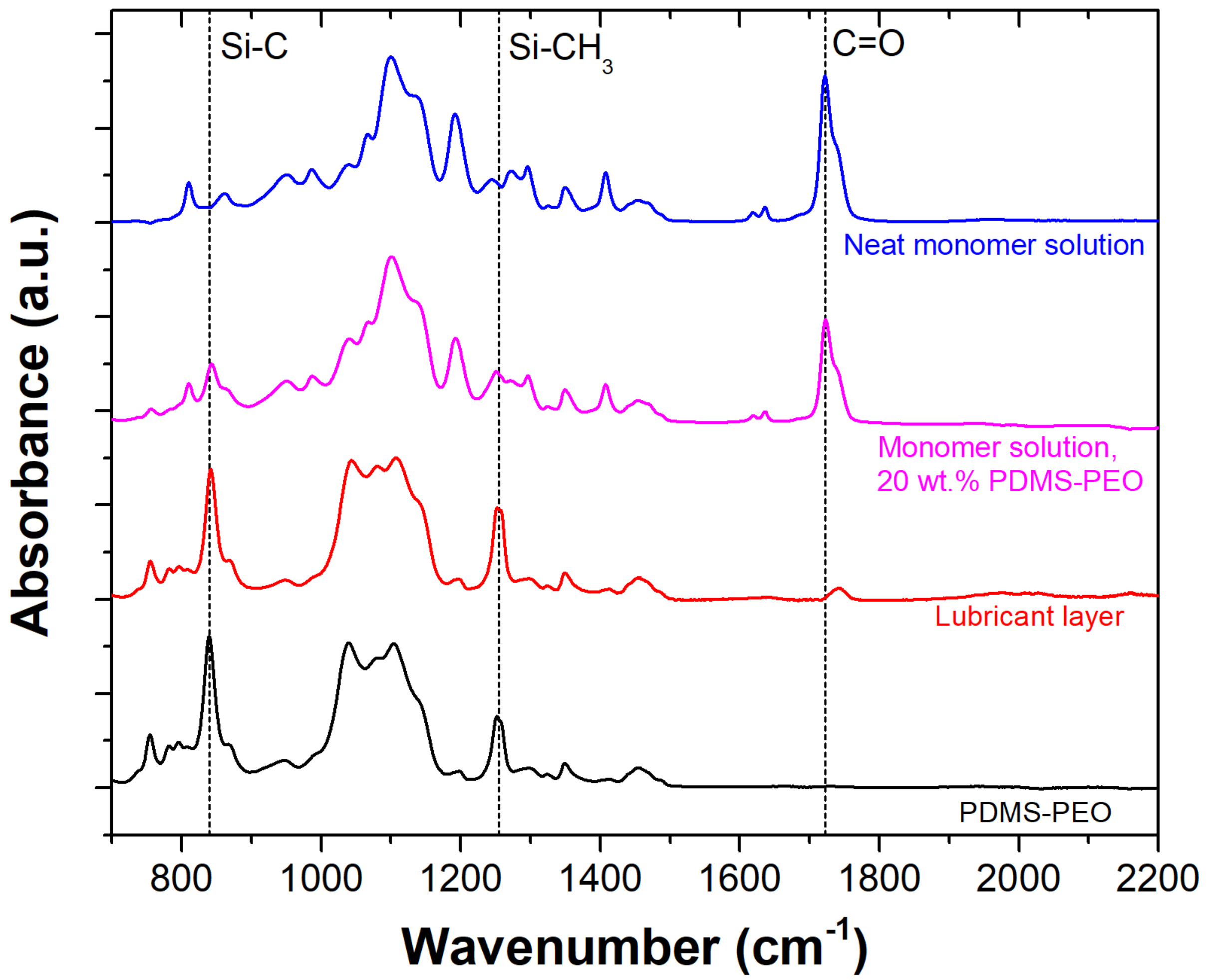

Figure S2. FTIR spectra of lubrication layer, monomer solution, and PDMS-PEO. The spectra for PDMS-PEO displays a characteristic Si-C rocking peak at 840 cm^{-1} and Si-CH_3 bending peak at 1255 cm^{-1}. The spectra for the monomer solution has a distinctive carbonyl (C=O) stretch at 1720 cm^{-1}. In the monomer solution containing 20 wt.% PDMS-PEO, both peaks are present. In the lubricant layer, the Si-C and Si-CH_3 predominate, with only a small peak near 1740cm^{-1} attributable to the monomer solution, indicating a lubricant layer composed of majority PDMS-PEO.

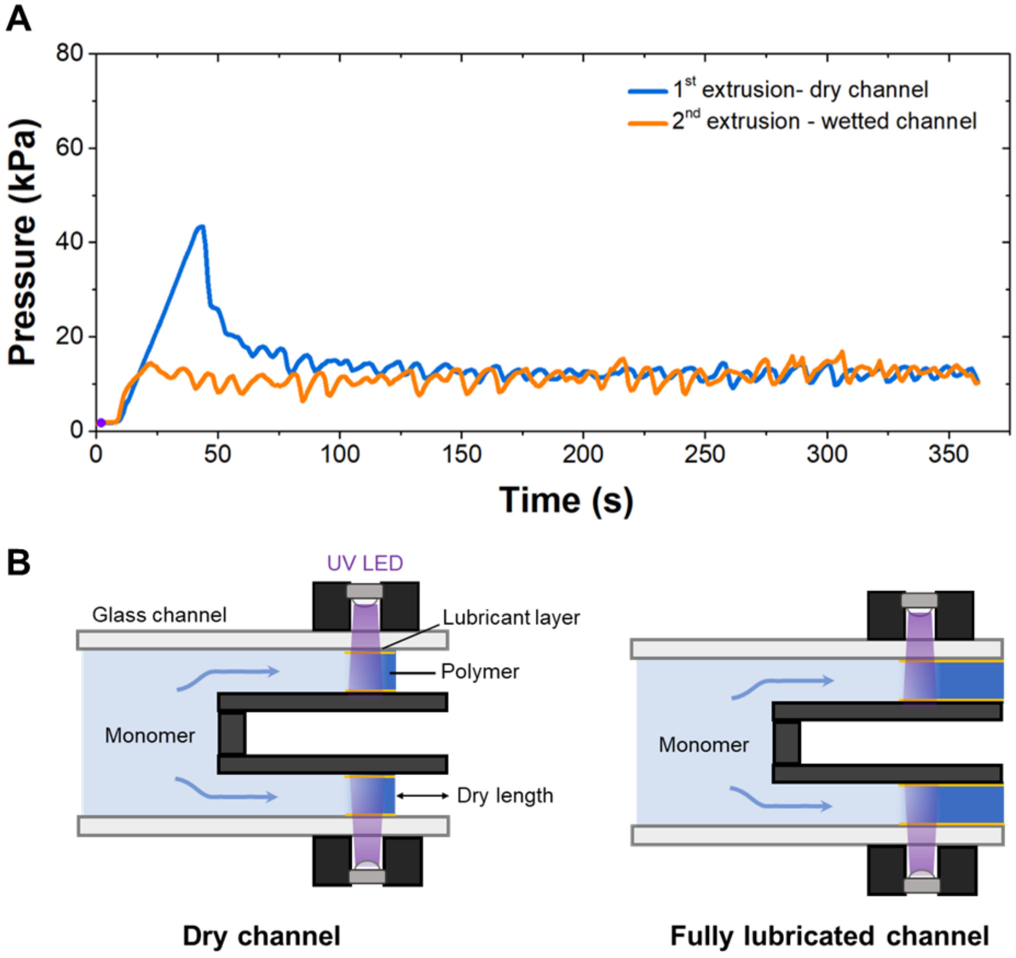

Figure S3. Extrusion behavior and development of lubrication layer. (a) Pressure over time for annular extrusion of monomer solution with 20 wt.% PDMS-PEO. The first extrusion is conducted in a cleaned, dry channel and features a large rise initial pressure in forming the lubrication layer. The subsequent extrusion occurs in an already wetted channel with the previous generated tube removed, with the fluid pressure reaching steady-state likely due to residual lubricant layer at the interface. **(b)** Schematic of extrusion in dry channel, with a 'dry' length that must be fully lubricated to reach continuous extrusion at a steady-state pressure.

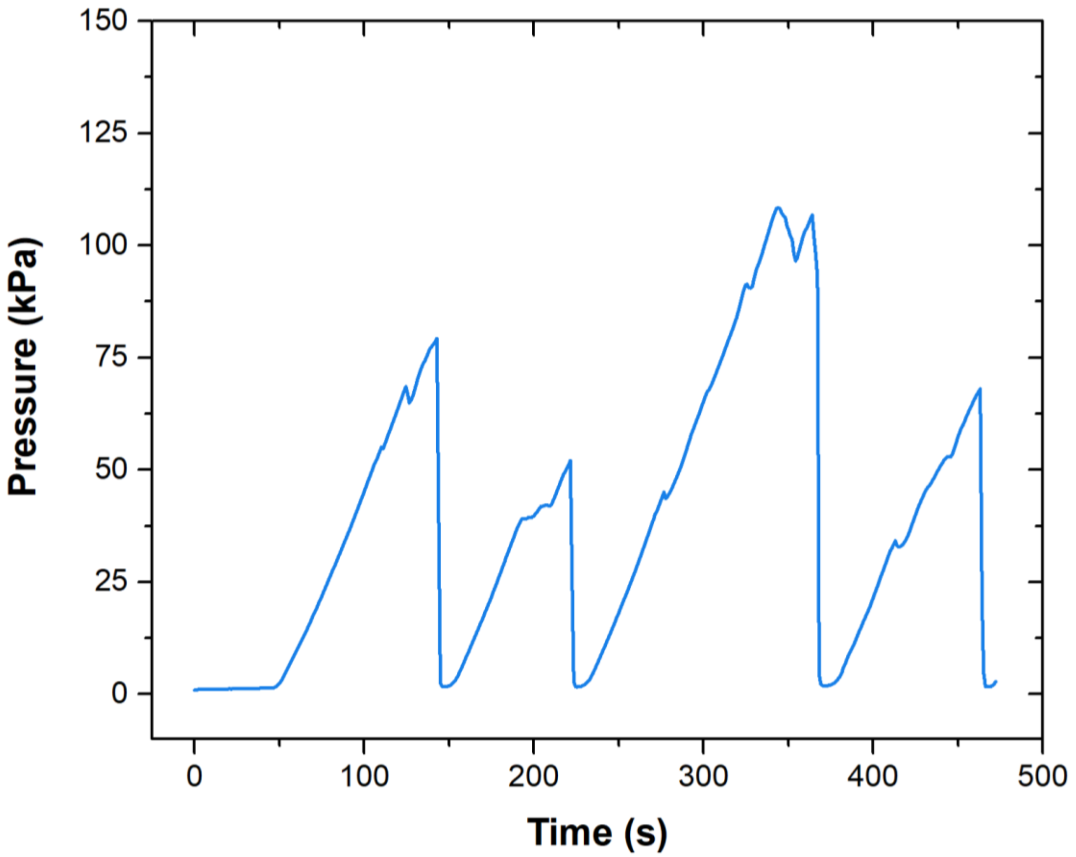

Figure S4. Fluid pressure during annular extrusion with untreated glass channel. Pressure data shows slip-stick events that result in the expulsion of the photopolymerized part from the channel and result in the large reduction of pressure. Pressure rises are associated with the formation of a new photopolymerization tube.

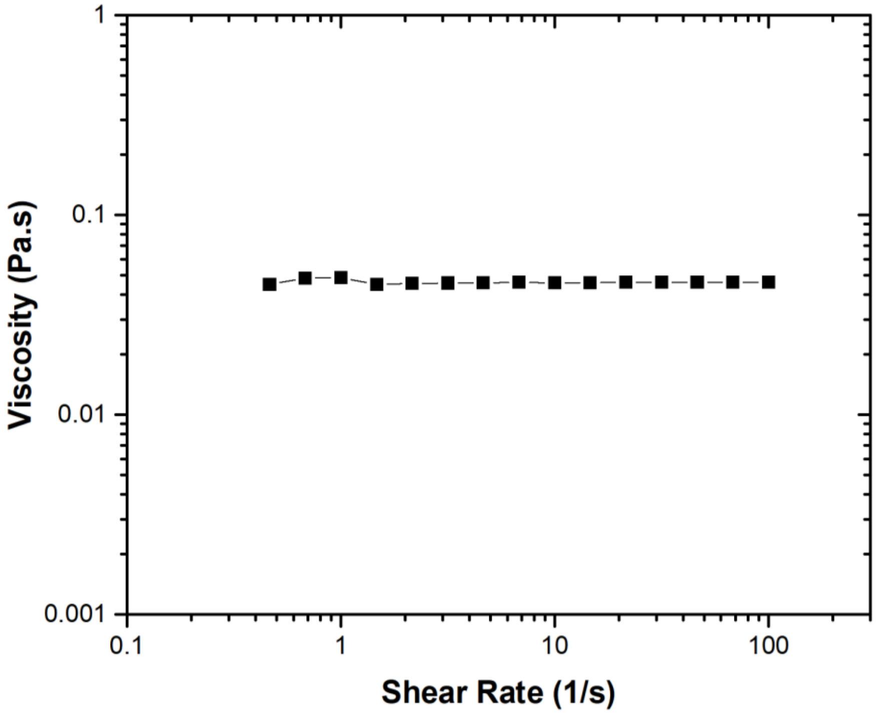

Figure S5. Shear rheology of monomer solution containing 20 wt.% PDMS-PEO.

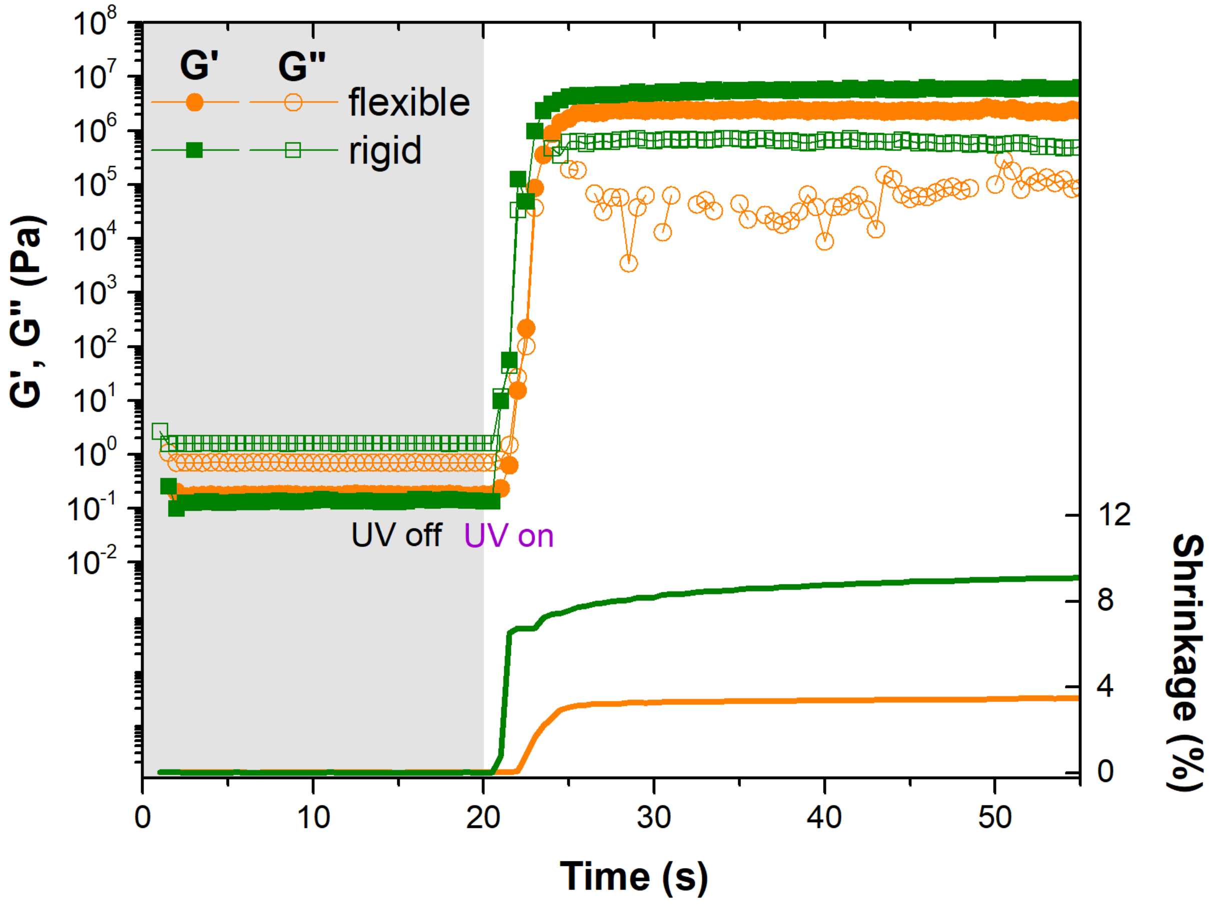

Figure S6. Photorheology of flexible and rigid thiol-ene resins with measured linear shrinkage.

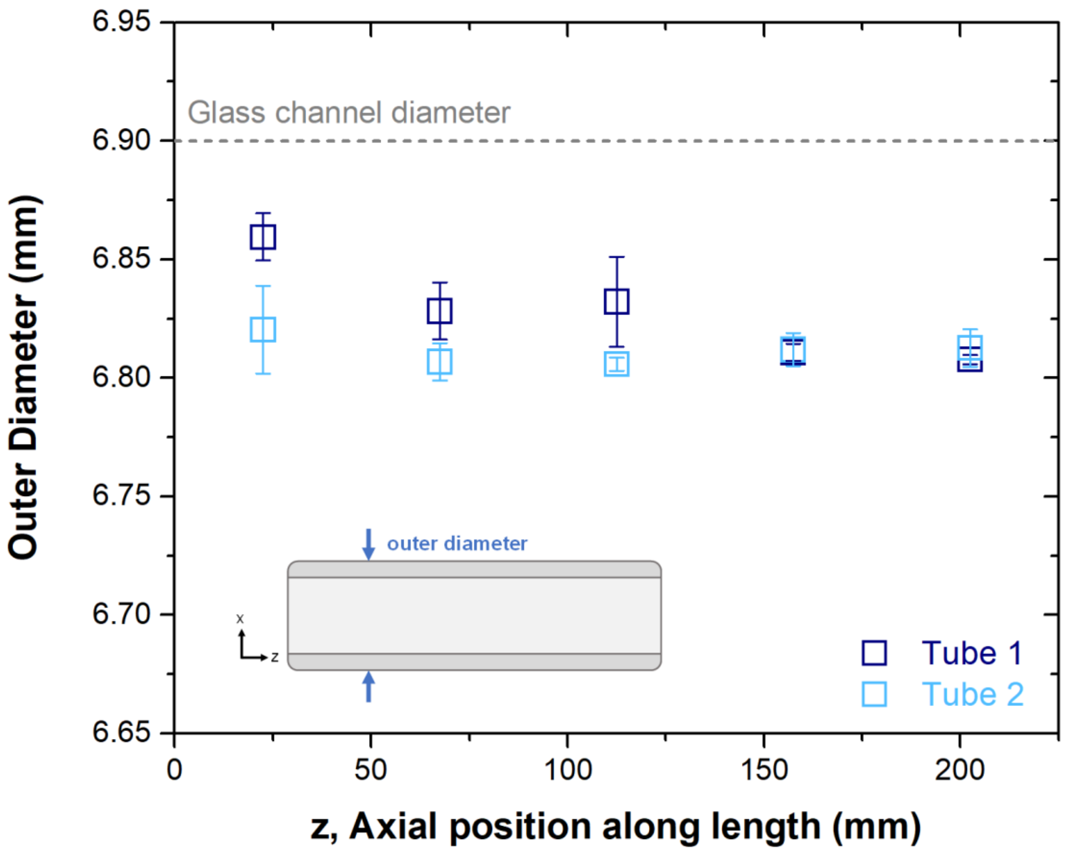

Figure S7. Average outer diameter of extruded tube as a function of axial length.

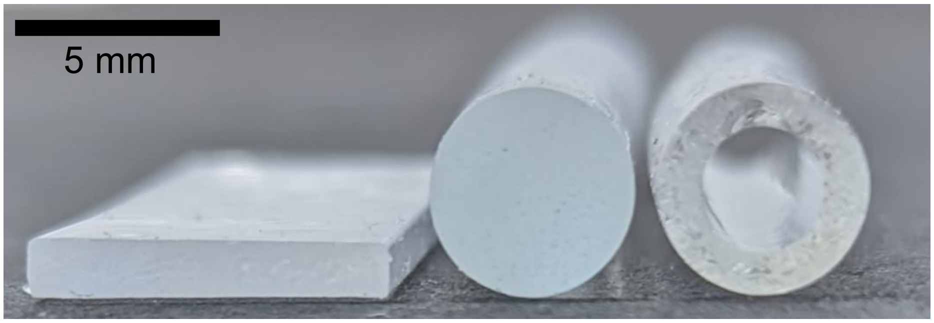

Figure S8. Different geometries can be extruded via E-SLIP. Rectangular, cylindrical and annular cross-sections can be extruded via the E-SLIP process.

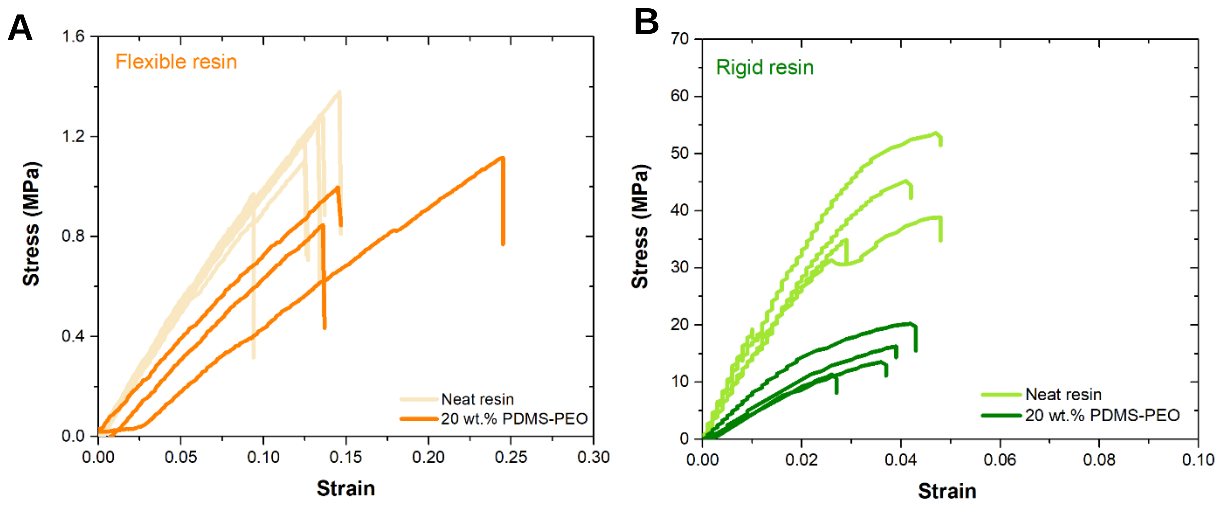

Figure S9. Mechanical properties of thiol-ene resins with and without 20 wt.% PDMS-PEO block copolymer. (a) Uniaxial tension stress-strain curves for flexible thiol-ene resins with and without PDMS-PEO. **(b)** Uniaxial tension stress-strain curves for rigid thiol-ene resins with and without PDMS-PEO.

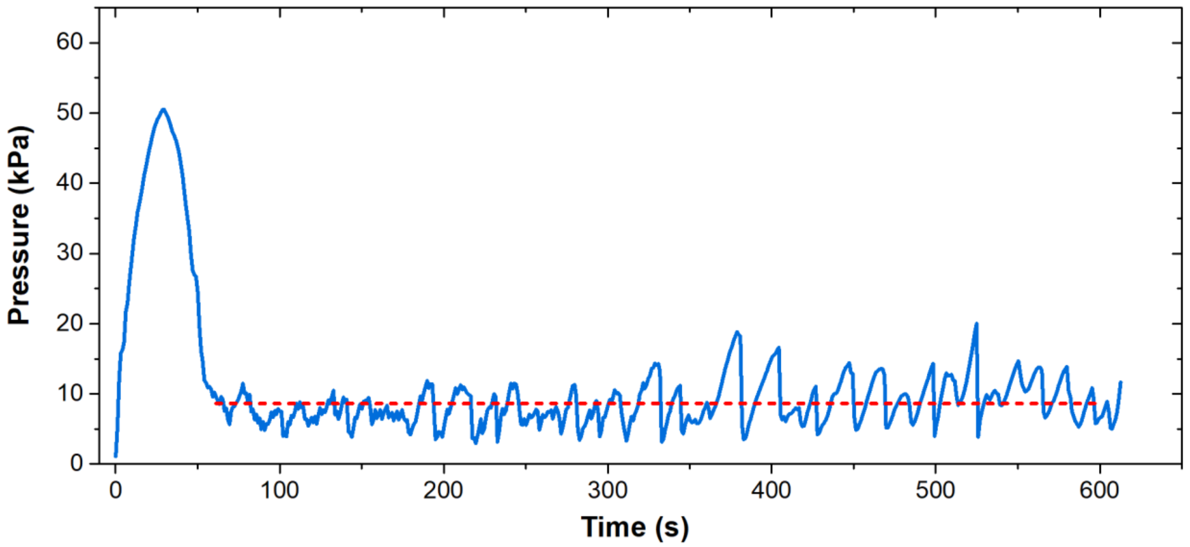

Figure S10. Pressure data for growing robot. Fluid pressure measured during a growing robot experiment with a flow rate of 1 mL/min is displayed in blue, with the time-averaged steady state pressure in dotted red.

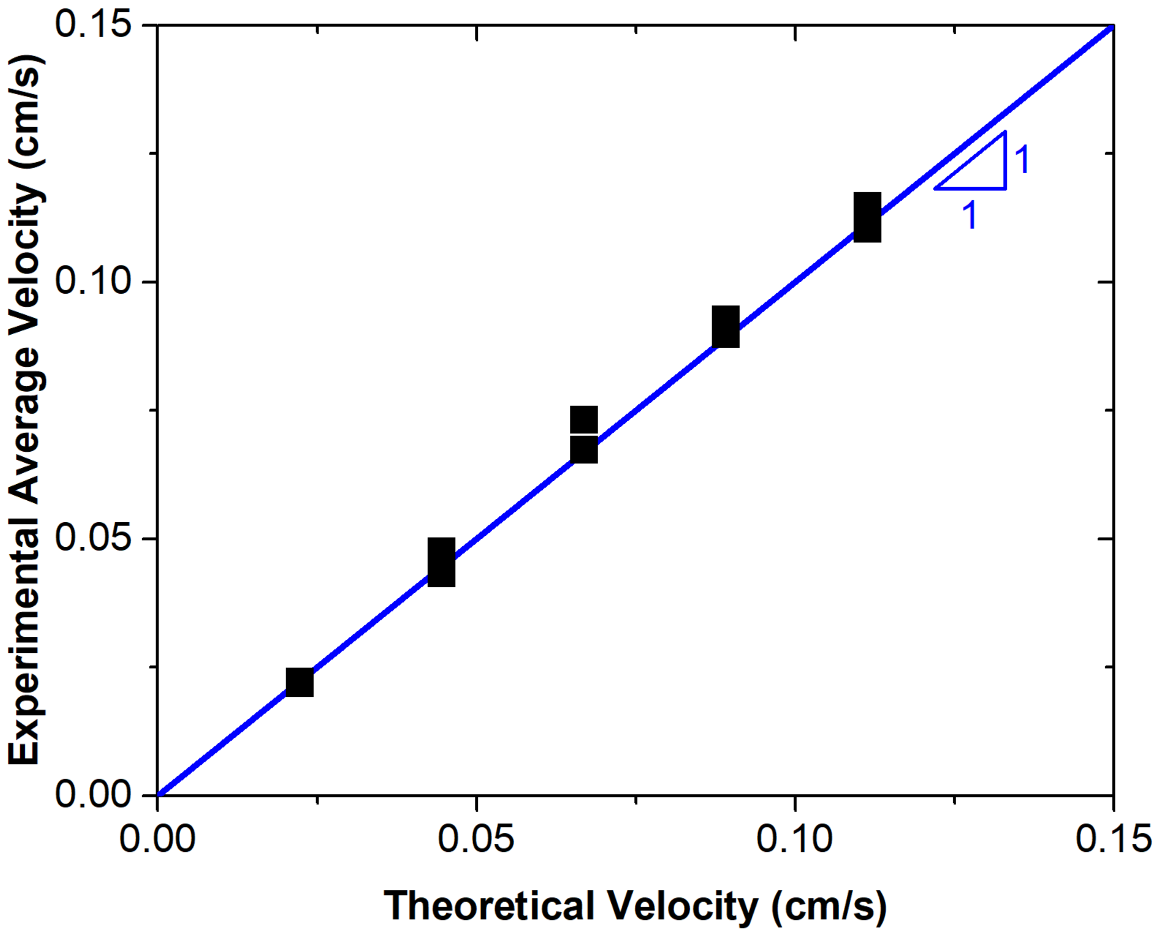

Figure S11. Robot velocity validation. A comparison of experimental average velocities calculated from length of tube grown and experiment time and the theoretical velocity based on the flow rate imposed by the syringe pump, with good approximate agreement demonstrated between the two.

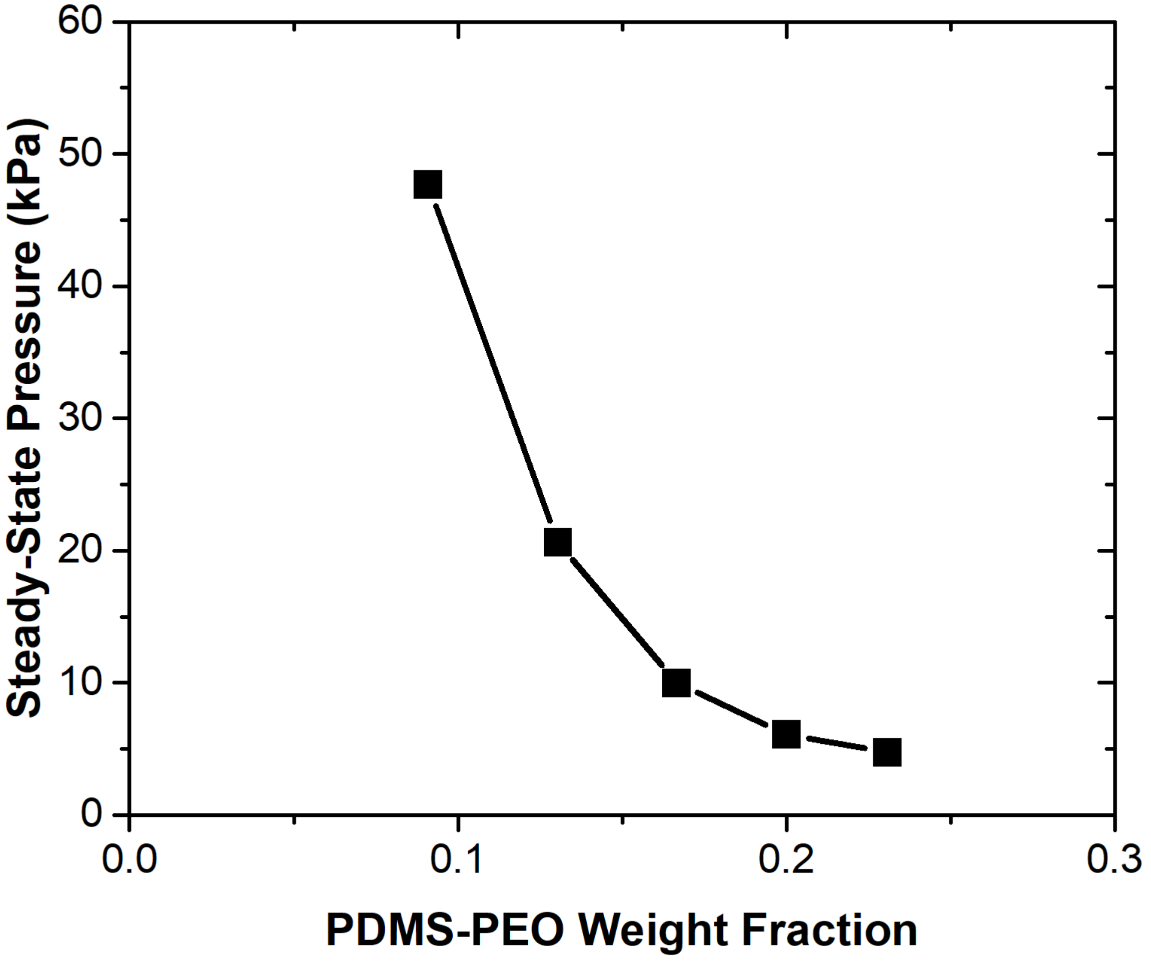

Figure S12. Lubrication in growing robot. Effect of PDMS-PEO block copolymer concentration on the steady-state pressure required for unimpeded horizontal growth in soft robot.

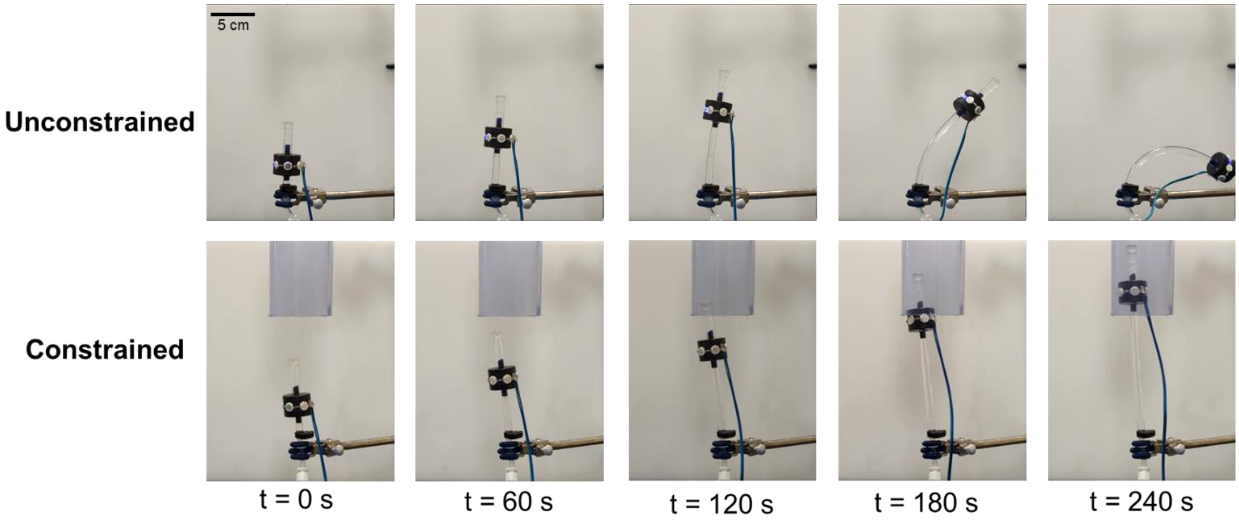

Figure S13. Unconstrained and constrained vertical growth with flexible resin. Unconstrained vertical growth eventually leads to buckling of the generated tube and a change of growth direction. Note that even though the tube buckles under this condition, the growth continues. Vertical growth can continue when robot is constrained inside a pipe due to prevention of tube buckling.

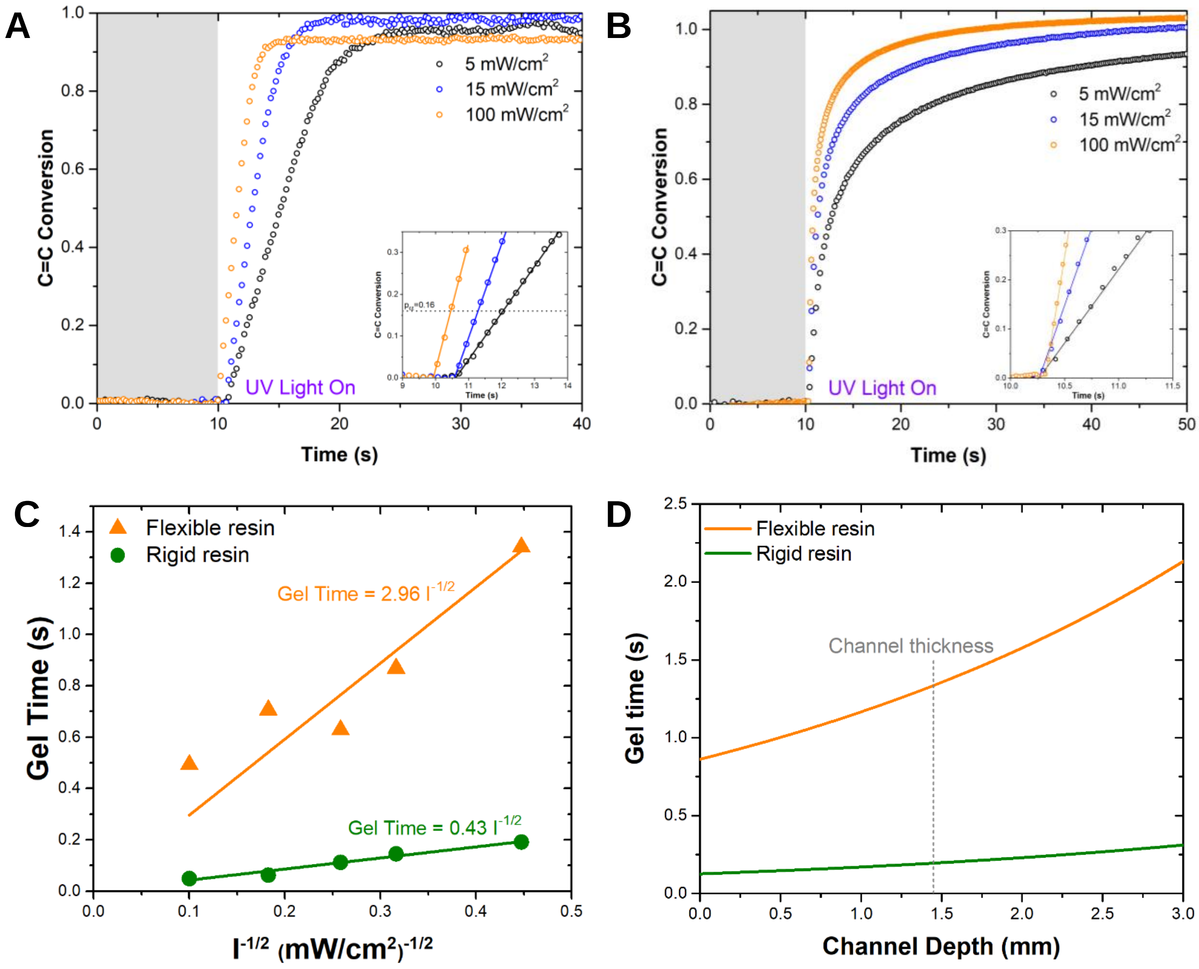

Figure S14. Photopolymerization characterization and depth of cure model. (a) Acrylate conversion of flexible resin with 20 wt.% PDMS-PEO during photopolymerization at several UV intensities, with linear fits in the low conversion regime to determine gel times. **(b)** Acrylate conversion of rigid resin with 20 wt.% PDMS-PEO during photopolymerization at several UV intensities, with linear fits in the low conversion regime to determine gel times. **(c)** Gel times of the flexible and rigid resins as a function of UV light intensity (*I*) determined from FTIR conversion study, with the solid-line representing the best fit line through all data points. **(d)** Theoretical plot of the gel time as a function of channel depth, with the robot's annular thickness noted, based on Beer-Lambert intensity decay and power-law fits.

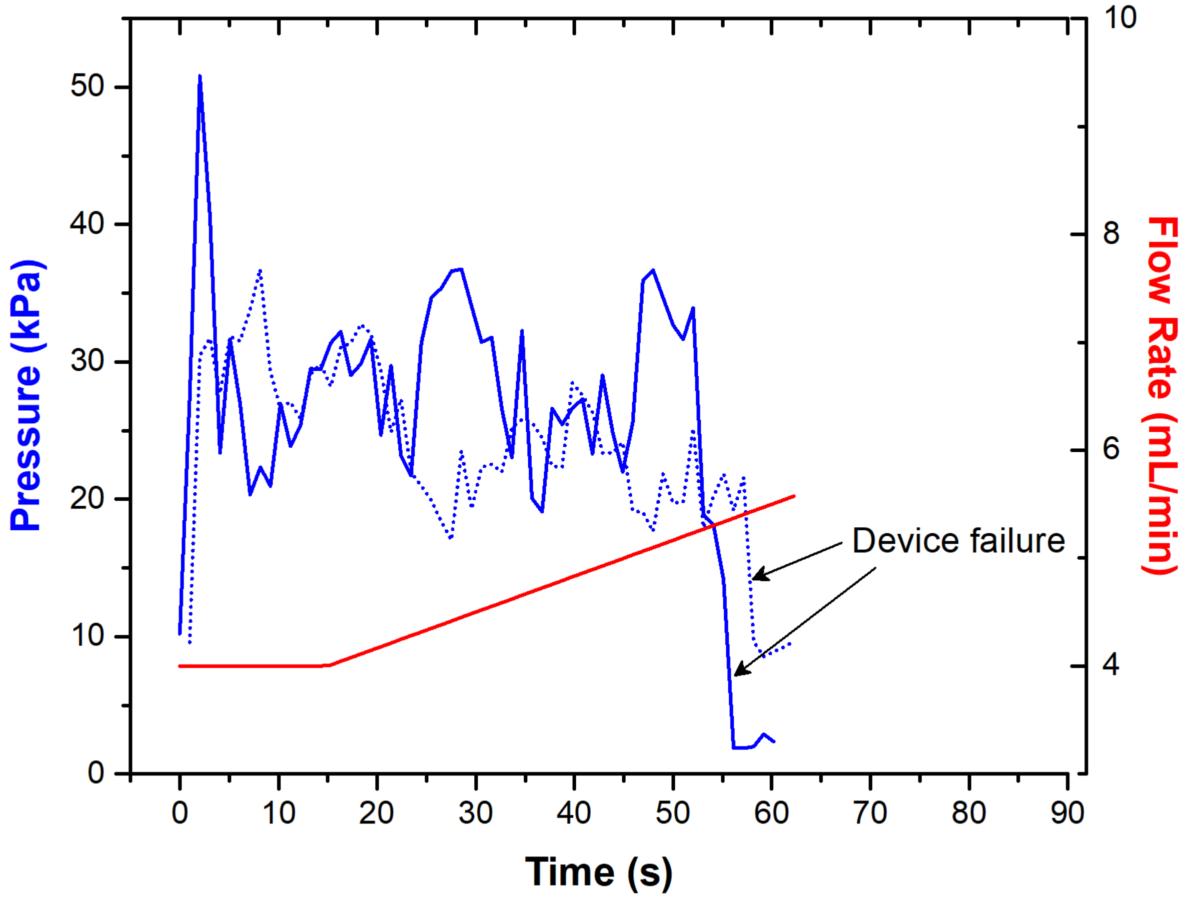

Figure S15. Flow rate ramps for experimental maximum velocity determination. Flow rates were linearly increased by 2 ml/min^2 to modulate robot velocity while the fluid pressure was measured, with two separate trials shown in solid and dotted lines. The decrease in fluid pressure demonstrates device failure due to incomplete curing of the photopolymerized tube, leading to monomer leakage. Experimental maximum velocities were calculated from the imposed flow rates at the point of tube failure according to the Equation S5.

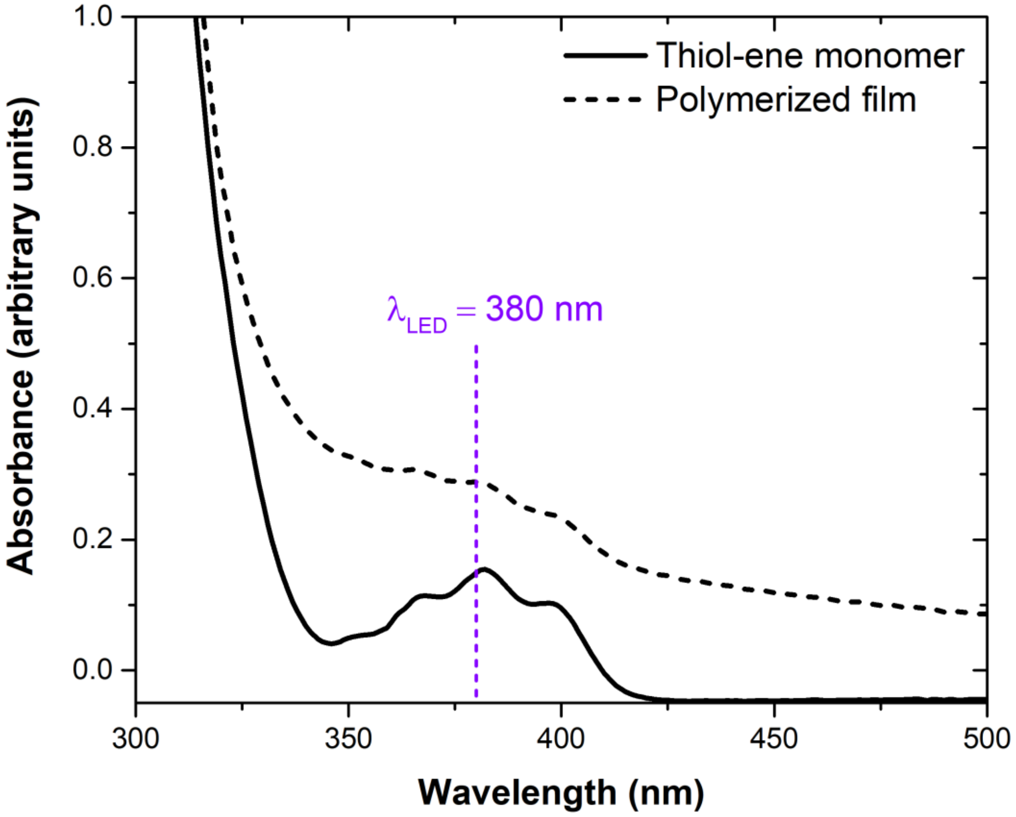

Figure S16. UV-visible absorption spectra of thiol-ene monomer and photopolymerized film. The polymerized film demonstrates increased absorption as compared to the thiol-ene monomer solution. This increase in absorption would result in further light attenuation during photopolymerization, leading to overestimation of the maximum theoretical velocity.

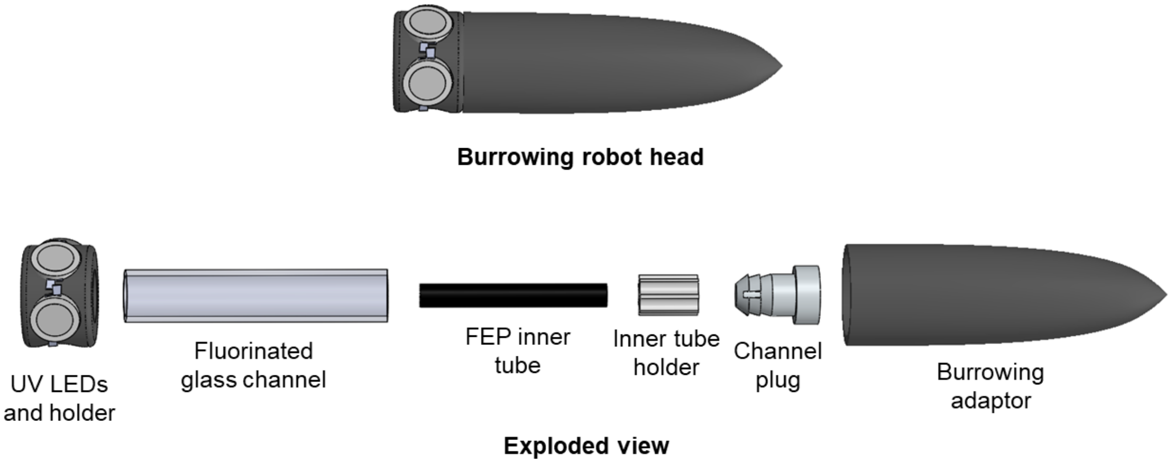

Figure S17. Schematic of robot head adapted for burrowing.

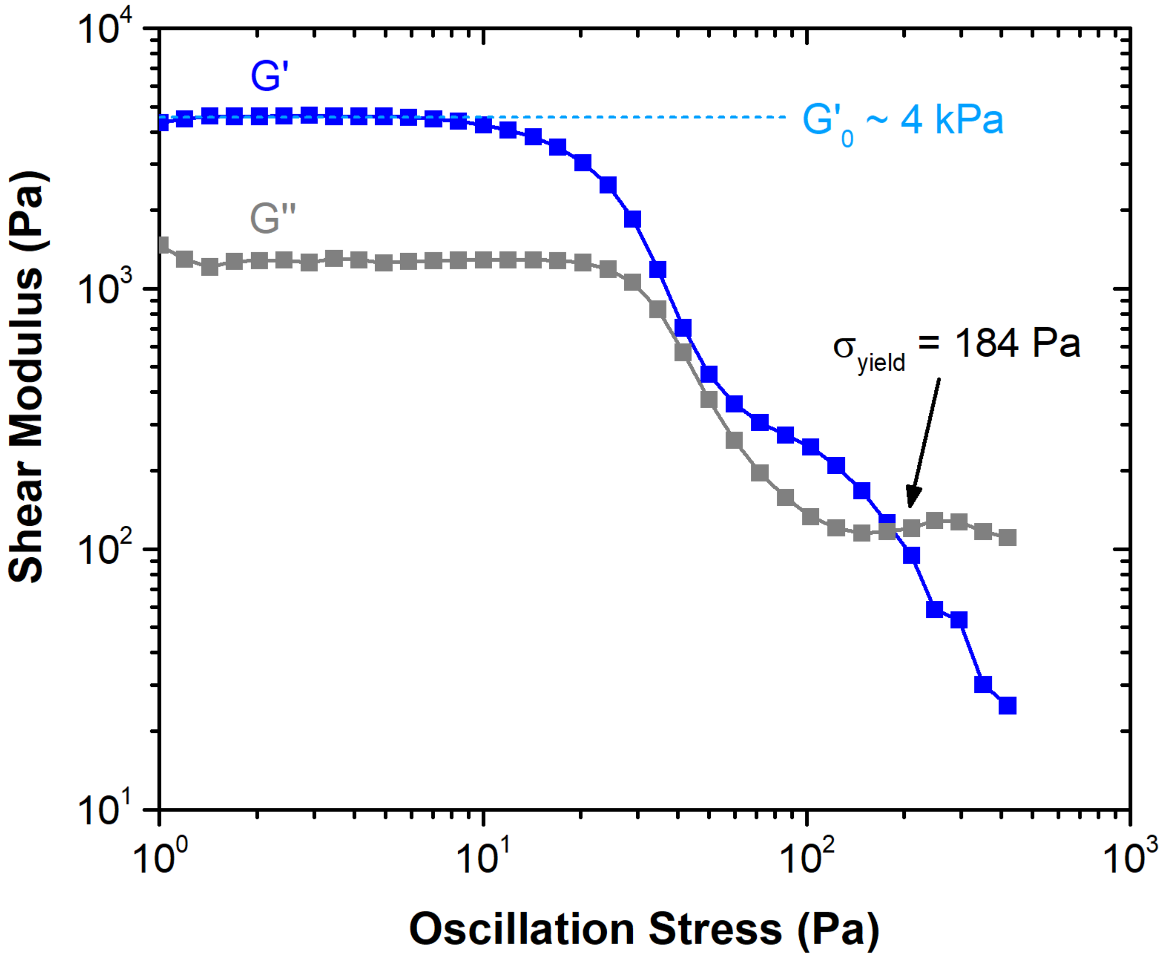

Figure S18. Oscillatory rheology of simulated soil. Oscillatory stress sweep of soil displaying a solid-like behavior with storage modulus (G') greater than the loss modulus (G") at low oscillation stresses. The plateau storage modulus (G'_0) and yield stress (σ_{yield}) (taken as the G' and G" crossover point) are provided in the figure.

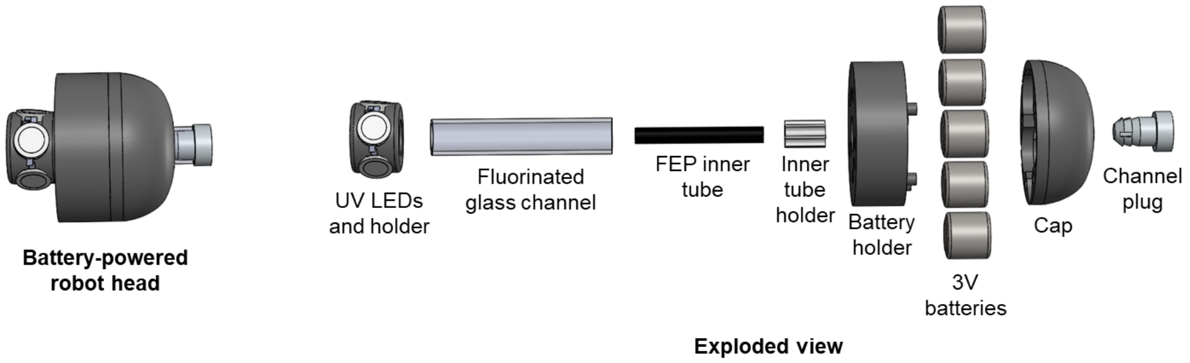

Figure S19. Schematic of battery-powered robot head.

Table S1. Mechanical properties of thiol-ene monomer resins utilized without and with 20 wt.% PDMS-PEO block copolymer.

Resin[a]	PDMS-PEO Wt. Frac. (%)	Modulus (MPa)	Tensile Strength (MPa)	Elongation at break (%)
1.6:0:1	0	10.8 ± 0.3	1.2 ± 0.1	13 ± 2
1.6:0:1	20	6.8 ± 0.8	1.1 ± 0.2	15 ± 5
0.6:1:1	20	14 ± 1	0.9 ± 0.1	6.0 ± 0.4
0.4:1.2:1	20	34 ± 3	1.9 ± 0.6	5.8 ± 2.2
0:1.6:1	0	1250 ± 250	37.7 + 10	4.7 ± 1.1
0:1.6:1	20	570 ± 120	15.3 ± 0.6	3.7 ± 0.7

a, Resins with differing molar ratios of functional groups of PEGDA:PETA:PETMP

Table S2. Additives screened for generating a lubricating layer during the E-SLIP process.

Additive (*Product name*)	Mol. Wt. (g/mol)	PEO Content (wt. %)	Miscible with Monomer	Successful Extrusion
Poly(ethylene glycol)	200	100	Yes	No
Silicone oil	--	0	No	No
PDMS-PEO-25 (*DBE-224*)	10,000	25	No	--
PDMS-PEO-30 (*DBE-311*)	800-1,200	30	No	--
PDMS-PEO-45 (*DBE-411*)	400-500	45	Yes (up to 10 wt.%)	--
PDMS-PEO-50 (*DBE-621*)	2,500	50	No	--
PDMS-PEO-65 (*DBE-712*)	600	65	Yes (up to 30 wt.%)	Yes

Movie S1.
Extrusion by self-lubricated interface photopolymerization (E-SLIP) process producing photopolymer part.

Movie S2.
Horizontally growing robot on a PTFE substrate.

Movie S3.
Passive obstacle avoidance of growing robot to reach a target.

Movie S4.
Burrowing behavior of growing robot in a transparent, simulated soil.

Movie S5.
Robot navigating tortuous path with a battery-powered light source.

References

1. M. G. Iskander, J. Liu, S. Sadek, Transparent Amorphous Silica to Model Clay. *J. Geotech. Geoenvironmental Eng.* **128**, 262–273 (2002).
2. A. Fredrickson, R. B. Bird, Non-Newtonian Flow in Annuli. *Ind. Eng. Chem.* **50**, 347–352 (1958).
3. S. K. Reddy, O. Okay, C. N. Bowman, Network development in mixed step-chain growth thiol-vinyl photopolymerizations. *Macromolecules* **39**, 8832–8843 (2006).
4. N. B. Cramer, C. N. Bowman, Kinetics of Thiol – Ene and Thiol – Acrylate Photopolymerizations with Real-Time Fourier Transform Infrared. 3311–3319 (2001).
5. A. Banerji, K. Jin, K. Liu, M. K. Mahanthappa, C. J. Ellison, Cross-Linked Nonwoven Fibers by Room-Temperature Cure Blowing and in Situ Photopolymerization. *Macromolecules* **52**, 6662–6672 (2019).
